# DNA-binding activity of PIF7 links phytochrome B signaling to plant responses to vegetation proximity

**DOI:** 10.1093/plphys/kiag602

**Published:** 2026-08-12

**Authors:** Marta Diaz-Lopez, Pedro Pastor-Andreu, Wenting Qin, Angela Sanchez-Garcia, Esteban Burbano-Erazo, Laura Valverde, Sandi Paulišić, Manuel Rodriguez-Concepcion, Jordi Moreno-Romero, Jaume F Martinez-Garcia

**Affiliations:** Institute for Plant Molecular and Cell Biology (IBMCP), CSIC-UPV, Edifici 8E, CPI, Campus UPV, València 46022, Spain; Centre for Research in Agricultural Genomics (CRAG), CSIC-IRTA-UAB-UB, Edifici CRAG, Campus UAB Bellaterra, Cerdanyola del Vallès, Barcelona 08193, Spain; Institute for Plant Molecular and Cell Biology (IBMCP), CSIC-UPV, Edifici 8E, CPI, Campus UPV, València 46022, Spain; Centre for Research in Agricultural Genomics (CRAG), CSIC-IRTA-UAB-UB, Edifici CRAG, Campus UAB Bellaterra, Cerdanyola del Vallès, Barcelona 08193, Spain; Institute for Plant Molecular and Cell Biology (IBMCP), CSIC-UPV, Edifici 8E, CPI, Campus UPV, València 46022, Spain; Institute for Plant Molecular and Cell Biology (IBMCP), CSIC-UPV, Edifici 8E, CPI, Campus UPV, València 46022, Spain; Institute for Plant Molecular and Cell Biology (IBMCP), CSIC-UPV, Edifici 8E, CPI, Campus UPV, València 46022, Spain; Centre for Research in Agricultural Genomics (CRAG), CSIC-IRTA-UAB-UB, Edifici CRAG, Campus UAB Bellaterra, Cerdanyola del Vallès, Barcelona 08193, Spain; Institute for Plant Molecular and Cell Biology (IBMCP), CSIC-UPV, Edifici 8E, CPI, Campus UPV, València 46022, Spain; Institute for Plant Molecular and Cell Biology (IBMCP), CSIC-UPV, Edifici 8E, CPI, Campus UPV, València 46022, Spain; Centre for Research in Agricultural Genomics (CRAG), CSIC-IRTA-UAB-UB, Edifici CRAG, Campus UAB Bellaterra, Cerdanyola del Vallès, Barcelona 08193, Spain; Departament de Bioquimica i Biologia Molecular, Universitat Autònoma de Barcelona, Campus UAB Bellaterra, Cerdanyola del Vallès, Barcelona 08193, Spain; Institute for Plant Molecular and Cell Biology (IBMCP), CSIC-UPV, Edifici 8E, CPI, Campus UPV, València 46022, Spain

## Abstract

PHYTOCHROME INTERACTING FACTORs (PIFs) are transcription factors that act as central signaling hubs in light-regulated processes. All PIFs contain an active phytochrome B–binding motif and a DNA-binding basic helix-loop-helix domain. In the shade-avoider *Arabidopsis thaliana*, PIF7 is a major promoter of hypocotyl elongation in response to vegetation proximity, becoming active when released from phytochrome B via its active phytochrome B–binding motif. Here we show that PIF7 promotes seedling elongation in other species, including the shade-avoider tomato and the shade-tolerant *Cardamine hirsuta*, suggesting that PIF7 has retained some of its key functional domains across diverse plants. Through complementation analyses using PIF7 variants lacking either the active phytochrome B–binding or basic helix-loop-helix domain, we demonstrate that, unlike PIF3, PIF7 versions unable to bind phytochrome B remain active regardless of light conditions, whereas loss of DNA-binding capacity fully disrupts PIF7 function. Our results further suggest that phytochrome B interaction imposes a dual regulatory control over PIF7, modulating both its abundance and its phosphorylation state (ie its ability to bind and regulate target genes).

## Introduction

When sensing the close proximity of other plants, shade-avoider species initiate a set of responses known as the shade avoidance syndrome (SAS) that together aim to help escape shade and anticipate light starvation. In *Arabidopsis thaliana*, hypocotyl elongation in seedlings is the best-characterized SAS response, although adult plants also display additional traits, including petiole elongation, leaf blade expansion, promotion of flowering time, and reduced seed number, that together constitute the SAS (Casal 2012; Martinez-Garcia and Rodriguez-Concepcion 2023; Roig-Villanova et al 2025).

SAS responses are initiated after perception of changes in the red (R) to far-red light (FR) ratio (R:FR) caused by vegetation proximity. When the plant is growing isolated and/or unshaded (ie without any nearby competitors), it receives light with a high R:FR. By contrast, when the plant is close to other plants, surrounding vegetation reflects specifically FR and produces a decrease in the R:FR perceived, termed *proximity shade*. When a plant is shaded by nearby vegetation, the overlying photosynthetic tissues absorb blue and R while transmitting FR. As a result, both photosynthetically active radiation (PAR, 400 to 700 nm) and the R:FR are reduced, resulting in a condition termed *canopy shade*. Both proximity and canopy shade display a reduced R:FR. In the laboratory, vegetation proximity or shade is mimicked by enriching white light (W) with FR (W + FR), a treatment known as *simulated shade* (Martinez-Garcia and Rodriguez-Concepcion 2023).

Changes in R:FR are detected by phytochromes, a group of photoreceptors that exist in a photoequilibrium of inactive (Pr) and active (Pfr) forms, with phytochrome B (phyB) having a major role. The R:FR ratio affects the balance between the 2 isoforms. Under high R:FR, the balance shifts toward the active Pfr form, which can bind to the PHYTOCHROME INTERACTING FACTORs (PIFs) (Leivar and Quail 2011) and inhibits their activity. Low R:FR shifts the photoequilibrium toward the inactive Pr form, which relieves the repression imposed on PIFs (Leivar et al 2008). PIFs were first identified in *A. thaliana* as transcriptional regulators of the basic helix-loop-helix (bHLH) family. The bHLH domain allows (i) dimerization via the helix-loop-helix motif and (ii) binding to specific DNA sequences via the basic domain. PIFs bind preferentially to a type of E-box (CANNTG) variant known as the PIF-binding E-box (PBE-box, CACATG and CATGTG) and G-box (CACGTG) motifs (Martinez-Garcia et al 2000; Toledo-Ortiz et al 2003; Hornitschek et al 2012; Zhang et al 2013). In addition to the bHLH domain, all known PIFs contain an active phytochrome B (APB)–binding region, necessary for binding to active phyB (Khanna et al 2004). PIF1 and PIF3 also contain an active phytochrome A–binding motif that participates in binding to active phyA (Shen et al 2008).

Among all PIFs characterized (PIF1–PIF8) (Cai and Huq 2025), genetic analyses in *A. thaliana* indicate that PIF1, PIF3, PIF4, and PIF5 (the so-called PIF quartet, PIFQ) have an important role in repressing seedling de-etiolation (hence promoting an etiolated phenotype). Another PIF with a well-known role is PIF7, a major player in promoting *A. thaliana* shade-induced hypocotyl elongation (Li et al 2012). Whereas PIFQ members are considered photolabile (their abundance is strongly reduced in light) (Lorrain et al 2008; Zhang et al 2013), PIF7 is comparatively photostable (Leivar et al 2008; Li et al 2012). Mechanistically, all PIFs interact with active phyB via their APB domain (Leivar et al 2008), which results in PIF phosphorylation and subsequent degradation and/or reduced biological activity (Cai and Huq 2025).

Structure–function studies on PIF3 have shown that its DNA-binding activity is required to modulate early and rapid light-dependent changes in gene expression but not the long-term promotion of hypocotyl elongation. Conversely, phyB binding through the APB domain is necessary for PIF3 to promote hypocotyl elongation but not to regulate early and rapid changes in gene expression (Al-Sady et al 2008). This suggests that DNA binding is not essential for full activity of PIF3 and/or that PIF3 can activate transcription without directly mediating phyB-induced transcriptional activation at target promoters. Whether this also applies to other PIFs, like PIF7, is unknown.

PIF7 activity is regulated by rapid phosphorylation in response to shade. Under W (high R:FR), PIF7 exists in both phosphorylated and dephosphorylated forms, whereas under shade (low R:FR), it is predominantly dephosphorylated (Li et al 2012; Willige et al 2021; Martinez-Vasallo et al 2024). Phosphorylation reduces its DNA-binding capacity at target gene promoters (Li et al 2012; Willige et al 2021), whereas phosphomimetic mutations of Ser139 and Ser141 into Asp abolish PIF7 biological activity (Huang et al 2018). PhyB-dependent phosphorylation and photobody localization of PIF7 are functionally linked and revoked by low R:FR to induce PIF7 activity (Willige et al 2021). Together, these findings support that dephosphorylated PIF7 is the active form promoting shade-induced hypocotyl elongation.

In this study, we investigate the molecular mechanism of PIF7 action. We show PIF7 promotes (i) elongation in light-grown seedlings of tomato and the shade-tolerant *Cardamine hirsuta* and (ii) shade-induced hypocotyl elongation not only in the shade-avoider *A. thaliana* but also in *C. hirsuta sis1* mutant seedlings (Molina-Contreras et al 2019). By complementing an *A. thaliana pif7* mutant with PIF7 variants, we further demonstrate that, unlike PIF3, preventing phyB binding results in shade-independent activity of PIF7, whereas loss of DNA binding fully disrupts PIF7 function. These findings unveil a conserved crosstalk of the phyB- and DNA-binding activities, advancing our understanding of PIF7’s role in shade regulation across plant species.

## Results

### PIF7 regulates responses to vegetation proximity in species other than *A. thaliana*

Comparison of the amino acid sequences of PIF7 from *A. thaliana* (AtPIF7), tomato (*Solanum lycopersicum*) (SlPIF7a, SlPIF7b), and *C. hirsuta* (ChPIF7) identified highly similar APB-binding and bHLH domains (Fig. S1) (Rosado et al 2016). To address whether the reported role of PIF7 in regulating responses to vegetation proximity in *A. thaliana* is conserved in other shade-avoider (tomato) and shade-tolerant (*C. hirsuta*) species, we obtained mutant lines in tomato and *C. hirsuta* using CRISPR-Cas9.

**Figure 1:**
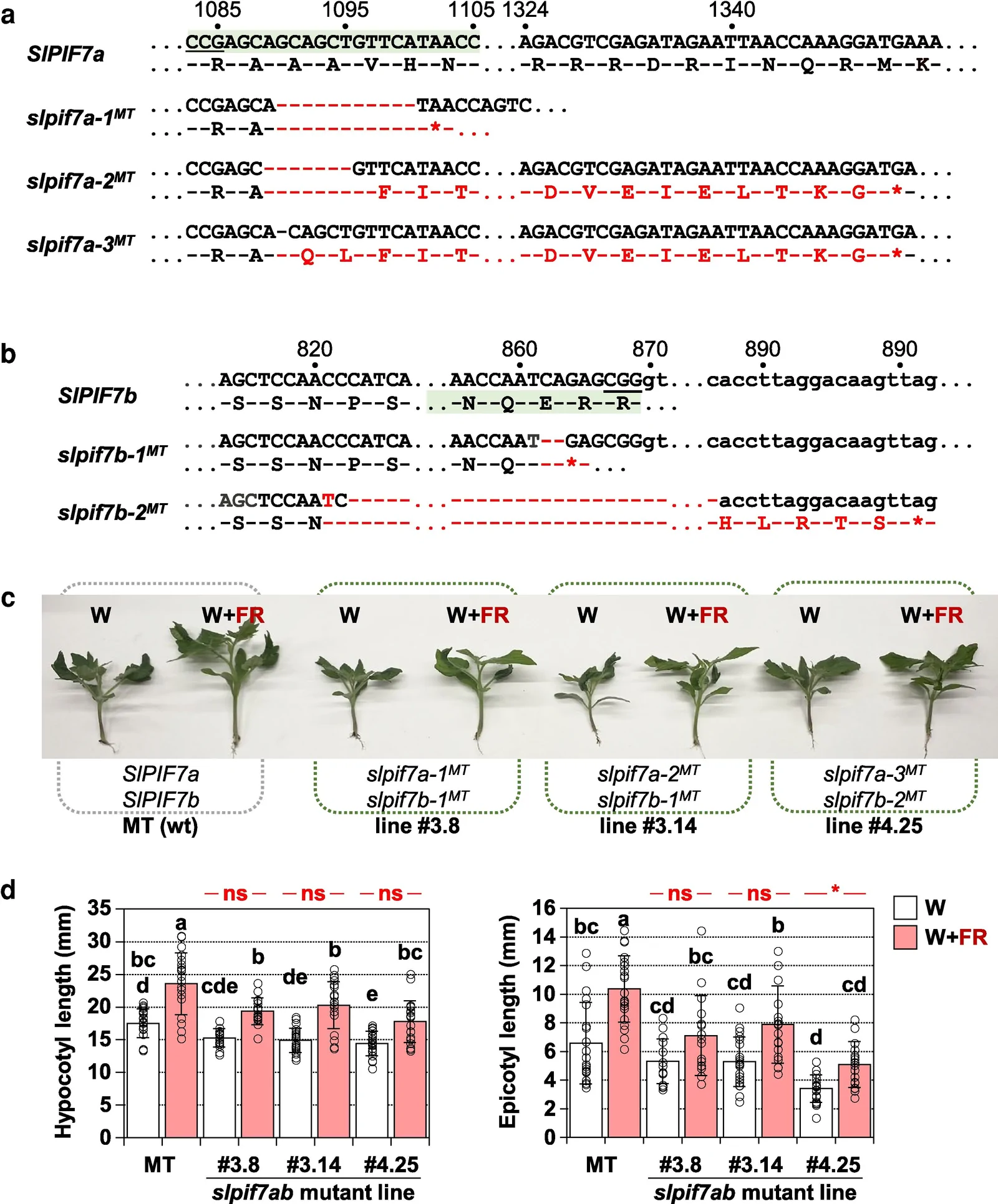
Seedlings of tomato lines deficient in *PIF7* show an attenuated response to simulated shade. Schematic representation of the mutant allele sequences of the a) *SlPIF7a* (*slpif7a-1^MT^*, *slpif7a-2^MT^*, and *slpif7a-3^MT^*) and b) *SlPIF7b* (*slpif7b-1^MT^* and *slpif7b-2^MT^*) genes identified, aligned with the corresponding wild-type MT genomic sequence (*SlPIF7a*, *SlPIF7b*). In a and b, the predicted amino acid sequence is shown below the corresponding DNA sequence. Nucleotide and amino acid differences relative to the wild-type are indicated in red. The light green box indicates the location of the gRNAs used for editing. The 3 nucleotides corresponding to the protospacer adjacent motif (PAM) are underlined. Nucleotides in lowercase refer to intron DNA. c) Aspect of representative tomato seedlings of MT and the double mutant *slpif7a slpif7b* (*slpif7ab*) lines #3.8, #3.14, and #4.25 grown in W and W + FR. The alleles of *SlPIF7a* and *SlPIF7b* present in each line are also indicated. Seedlings were germinated and grown in darkness for 4 d, then grown under W for 2 d and exposed to W + FR or kept under W for 11 d. d) Hypocotyl and epicotyl length of MT and *slpif7ab* mutant lines grown in W (white bars) and W + FR (pink bars) conditions, as indicated in c. Values are the mean ± SD of 16 to 21 seedlings per genotype and treatment (shown as empty black dots). Different letters above bars indicate statistically significant differences (1-way ANOVA followed by Tukey's test, *P* < 0.05) among means. Red asterisks indicate significant differences (2-way ANOVA, *P* < 0.05; ns, not significant) of the response to simulated shade of each double mutant *slpif7ab* line compared to MT.

We identified 3 tomato lines having mutations in both *SlPIF7a* and *SlPIF7b* genes in the MicroTom (MT) background: #3.8 (with *slpif7a-1^MT^*, containing an 11-bp deletion, and *slpif7b-1^MT^*, with a 2-bp deletion), #3.14 (with *slpif7a-2^MT^*, containing a 7-bp deletion, and *slpif7b-1^MT^*, the same allele as in line #3.8), and #4.25 (with *slpif7a-3^MT^*, containing a 1-bp deletion, and *slpif7b-2^MT^*, containing a 64-bp deletion and an adjacent C to T transition) (Fig. 1a and b). All these mutations changed the open reading frame and resulted in early stop codons that interrupted the basic domain of the bHLH, likely producing inactive SlPIF7a and SlPIF7b proteins (Fig. 1a and b).

Six-day-old W-grown seedlings of tomato MT (wild type) and the 3 PIF7-defective mutant lines were germinated in darkness; evenly grown 4-d-old seedlings were transferred to soil and incubated under W for 2 additional days. Then, half were grown under W or W + FR for 11 additional days (Fig. S2a). At the end of the treatment, hypocotyl and epicotyl (first internode) lengths were measured as indicative of the seedling shade response (Burbano-Erazo et al 2025). Both the hypocotyls and epicotyls of the mutant lines were slightly shorter than those of the MT line under W (Fig. 1c and d), suggesting a role for both *SlPIF7* genes in controlling seedling length under our long-day (LD) photoperiodic conditions (16 h W, 8 h darkness). Additionally, all 3 mutant lines displayed attenuated hypocotyl and epicotyl elongation under W + FR compared with the MT parental line (Fig. 1d). These results suggest that *SlPIF7a* and *SlPIF7b* promote elongation under both W and W + FR. The difference in hypocotyl and epicotyl length in W + FR and W (HYP_W_  _+_  _FR_-HYP_W_ and EPI_W_  _+_  _FR_-EPI_W_) was estimated to visualize the elongation response to shade (Fig. S2b and c), showing that *slpif7a slpif7b* double mutants responded similarly to the MT line. Similar conclusions were reached when seedlings were germinated in W (Fig. S2d–f). In summary, these results support that SlPIF7a and SlPIF7b have a role in promoting hypocotyl and epicotyl length under both W and W + FR in tomato MT seedlings but not in the elongation response to shade. Therefore, the tomato shade-induced elongation response involves the participation of additional components, an interpretation consistent with the reported role of *SlPIF8a* in regulating this trait in a different tomato cultivar (Kunta et al 2025).

**Figure 2:**
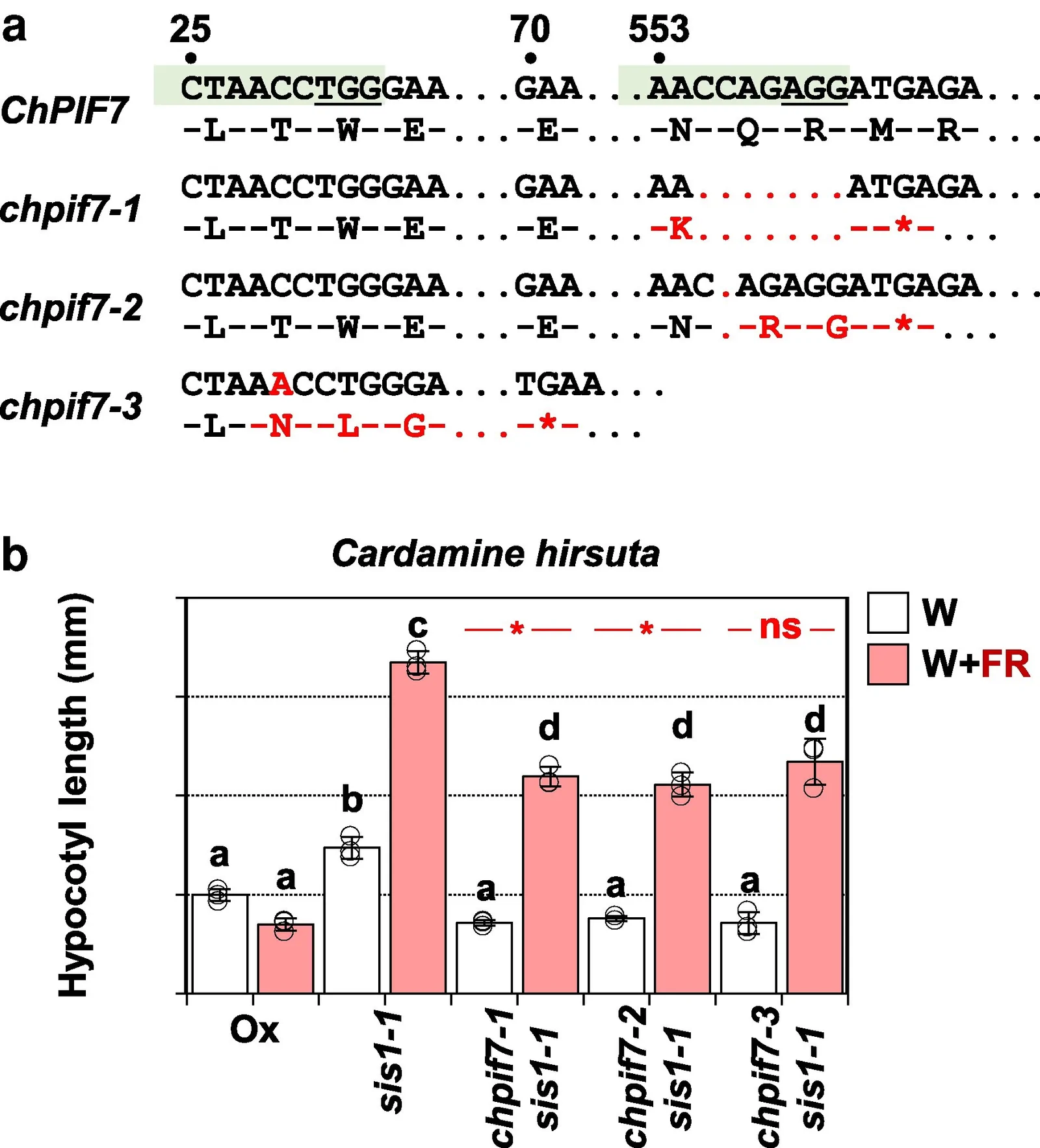
Seedlings of *C. hirsuta sis1* mutant lines deficient in *PIF7* show an attenuated response to simulated shade. a) Schematic diagram showing the mutations found in the *ChPIF7* gene of the different mutant alleles (*chpif7-1*, *chpif7-2*, and *chpif7-3*) compared with the wild-type sequence (ChPIF7) and the predicted amino acid changes. The light green box indicates the location of the gRNAs used for editing. The 3 nct corresponding to the PAM are underlined. b) Hypocotyl length of Ox, *sis1-1* single mutant, and *sis1-1 chpif7-1*, *sis1-1 chpif7-2*, and *sis1-1 chpif7-3* double mutants. Seedlings were germinated for 3 d in W and then maintained in W or transferred to simulated shade (W + FR) for 4 more days, as represented in Fig. S2. Values are the mean ± SD of 3 independent biological replicates (shown as empty black dots). Each biological replicate is the mean of ca. 25 seedlings. Different letters denote significant differences (1-way ANOVA with the Tukey test, *P* < 0.05) among means. Red asterisks indicate significant differences (2-way ANOVA, *P* < 0.05; ns, not significant) in the response to simulated shade of each double *chpif7 sis1-1* mutant line compared to *sis1-1*.

We next edited *ChPIF7* in the shade-tolerant *C. hirsuta*. In this species, hypocotyls do not elongate under W + FR (Molina-Contreras et al 2019; Paulisic et al 2021), which complicates the assessment of *ChPIF7* knockout effects. However, seedlings of the *slender in shade 1* (*sis1*) mutant, deficient in *ChPHYA*, exhibit strong hypocotyl elongation in response to W + FR (Molina-Contreras et al 2019). Given the reduced shade-induced hypocotyl elongation observed in *A. thaliana pif7 phyA* double mutants compared to *phyA* (Pastor-Andreu et al 2024), we hypothesized a similar effect in *C. hirsuta*. Therefore, we edited *ChPIF7* in the *sis1-1* mutant background. We obtained 3 independent *chpif7* mutant alleles with either a 7-bp (*chpif7-1*) or 1-bp (*chpif7-2*) deletion or a 1-bp insertion (*chpif7-3*), all of which disrupted the open reading frame, introducing premature stop codons and leading to truncated ChPIF7 proteins (Fig. 2a). As expected, wild-type (Ox) seedlings showed no statistical difference in hypocotyl length between W and W + FR conditions. In contrast, the *sis1 chpif7* double mutants displayed an attenuated elongation response to shade compared to *sis1-1* hypocotyls, which strongly elongated under the same conditions (Fig. 2b and Fig. S3b). After crossing these lines with Ox to segregate away the *sis1* mutation, we observed that *chpif7* single mutant seedlings did not elongate under W + FR, behaving like wild-type seedlings, as expected. However, these seedlings were significantly shorter than Ox in both W and W + FR (Fig. S3c), a phenotype consistent with promoting elongation of SlPIF7a and SlPIF7b in tomato seedlings growing in W and W + FR (Fig. 1d, Fig. S2b and c), as well as PIF7 in *A. thaliana* seedlings growing under short-day photoperiodic conditions (Leivar et al 2020). Importantly, as in *A. thaliana*, PIF7 promotes the elongation response to shade in *C. hirsuta*, an effect that becomes evident only in the absence of phyA, when these seedlings have recovered the capacity to elongate in response to simulated shade.

### Transgenic lines expressing different PIF7 derivatives differ in their shade-induced biological activity

The observation that some aspects of the biological role of PIF7 are conserved in plant species beyond *A. thaliana*, including those with divergent strategies to vegetation proximity, is consistent with the conservation of the core features of PIF7, namely, the APB and bHLH motifs (Fig. S1a). Indeed, the invariant amino acid EL and GQ residues, required for APB function in *A. thaliana* (Khanna et al 2004), are conserved also in ChPIF7, SlPIF7a, and SlPIF7b (Rosado et al 2016) and even in SlPIF8a, reported as having a major role in the shade-induced promotion of elongation in tomato cultivar M82 (Kunta et al 2025), ChPIF8, and AtPIF8 (Fig. S1b). The bHLH region is also conserved between the PIF7 and PIF8 sequences, including the H-E-R signature in the basic domain (positions 5-9-13), required for DNA binding and present in the DNA-binding bHLHs, and positions 10 and 12, in direct contact with the DNA backbone (Galstyan et al 2011; Galstyan et al 2012) (Fig. S1c).

**Figure 3:**
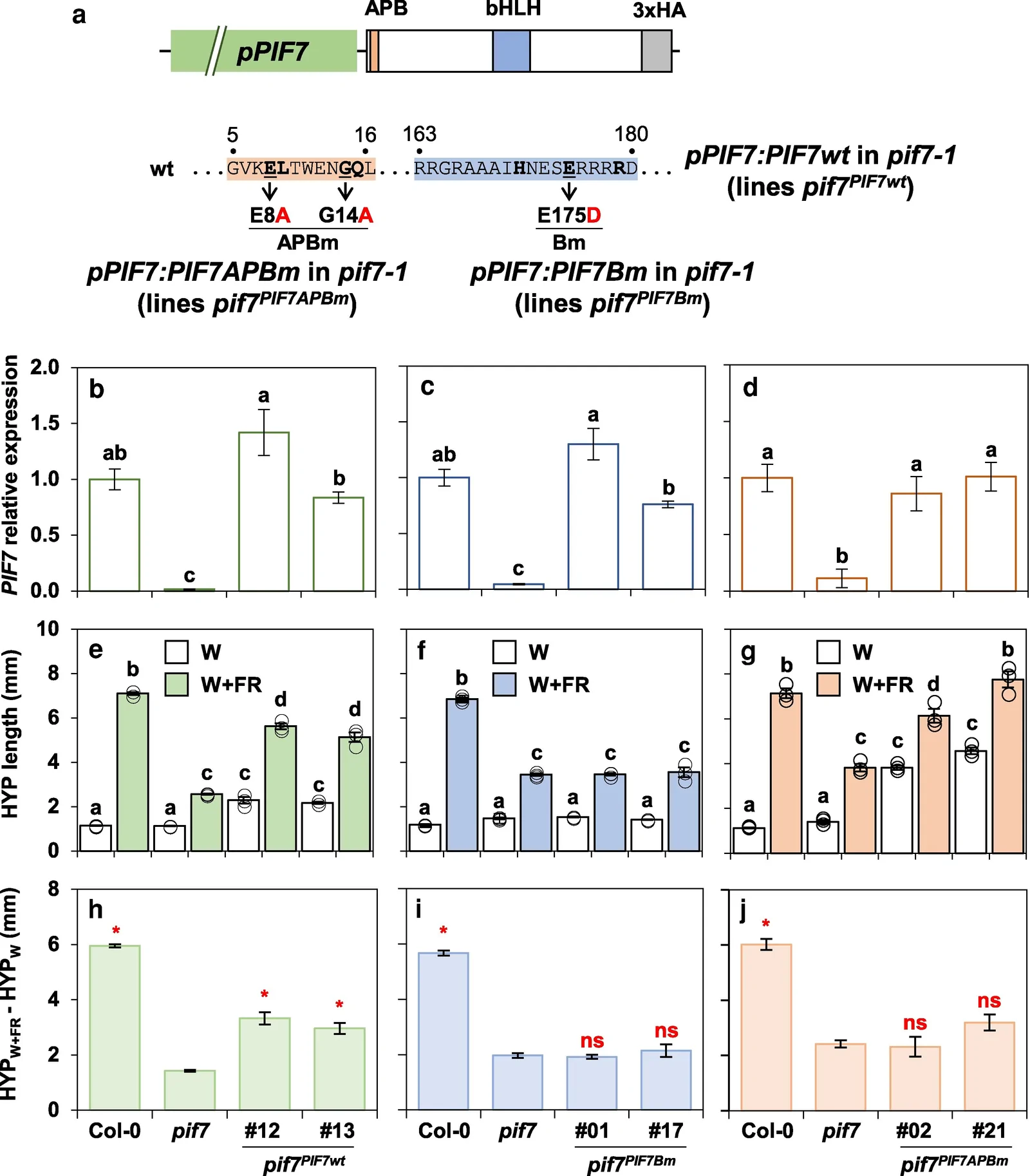
Mutant Arabidopsis PIF7 derivatives display different activity in the hypocotyl response to simulated shade of *A. thaliana* seedlings. a) Cartoon depicting the generated PIF7 derivatives with different mutations used to transform *pif7-1*. The APB domain is highlighted in light brown and the bHLH in blue. Mutated amino acids are highlighted in red. *pPIF7*, endogenous promoter of *PIF7* driving the expression of the different *PIF7* variants. Relative *PIF7* transcript in seedlings of Col-0 and *pif7-1* and b) *pif7^PIF7wt^*, c) *pif7^PIF7Bm^*, and d) *pif7^PIF7APBm^* lines, respectively. Seedlings were grown 7 d in W. Hypocotyl (HYP) length in W and W + FR of seedlings of Col-0 and *pif7-1* compared with e) *pif7^PIF7wt^*, f) *pif7^PIF7Bm^*, and g) *pif7^PIF7APBm^* lines, respectively. Difference in hypocotyl length in W + FR and W for Col-0 and *pif7-1* and h) *pif7^PIF7wt^*, i) *pif7^PIF7Bm^*, and j) *pif7^PIF7APBm^* lines, respectively. In e to j, seedlings were germinated for 2 d in W and then maintained in W or transferred to simulated shade (W + FR) for 5 more days, as represented in Fig. S4. In e to g, values are the mean ± SE of 3 independent biological replicates (shown as empty black dots). Each biological replicate is the mean of ca. 25 seedlings. Different letters denote significant differences (1-way ANOVA with the Tukey test, *P* < 0.05) among means. Red asterisks indicate significant differences (2-way ANOVA, *P* < 0.05; ns, not significant) in the response to simulated shade of Col-0 and transgenic lines compared to *pif7-1*.

To test the functionality of these conserved domains in response to simulated shade, we next performed a structure–function analysis on *AtPIF7*. Based on the location of the APB and the bHLH domains of AtPIF7 (Fig. S1, Fig. 3a) (Roig-Villanova et al 2007; Leivar et al 2008), we cloned and prepared 3 *AtPIF7* derivative constructs, using PCR-based mutagenesis on complementary DNA (cDNA) as a DNA template: a wild-type form (*PIF7wt*), an *AtPIF7* variant with the APB domain mutated (*PIF7APBm*), and an *AtPIF7* derivative with the bHLH domain mutated (*PIF7Bm*) (Fig. 3a). The introduced mutations in the sequences were chosen based on the amino acid changes previously demonstrated to abolish PIF1 and PIF3 binding to DNA (Al-Sady et al 2008; Shen et al 2008) and PIF7 interaction with phyB (Leivar et al 2008). In particular, mutations introduced in PIF7APBm changed Glu in position 8 (Glu8) and Gly14 for 2 Ala (E8AG14A), and those introduced in PIF7Bm substituted Glu175 for an Asp (E175D) (Fig. 3a). These *PIF7*-derivative constructs were fused to a triple hemagglutinin tag (3xHA) in the C-terminal end expressed under the control of a fragment of about 2 Kb (1,961 bp) of the *AtPIF7* promoter (*pPIF7*) (Fig. 3a).

The *A. thaliana pif7-1* mutant (in Col-0 background) was transformed with the resulting constructs, producing lines *pif7^PIF7w^*^t^ (*pPIF7:PIF7wt-3xHA*), *pif7^PIF7APBm^* (*pPIF7:PIF7APBm-3xHA*), and *pif7^PIF7Bm^* (*pPIF7:PIF7Bm-3xHA*) (Fig. 3a). We obtained 11, 8, and 5 independent T1 lines, with only 1 transfer DNA (T-DNA) insertion (T2 generation) expressing *PIF7wt*, *PIF7APBm*, and *PIF7Bm* derivatives, respectively, that were brought to homozygosis in the T3 generation. The relative *PIF7* expression levels in each line were estimated in seedlings grown for 7 d in W. For each construct, 2 lines were selected because transgenic *PIF7* expression levels were comparable to those of endogenous *PIF7* in Col-0: lines #12 and #13 for *pif7^PIF7wt^*, lines #02 and #21 for *pif7^PIF7APBm^*, and lines #01 and #17 for *pif7^PIF7Bm^* (Fig. 3b–d). As expected, *PIF7* transcript was virtually undetected in *pif7* mutant seedlings (Fig. 3b–d).

To functionally characterize the different PIF7 derivatives, seedlings of Col-0, *pif7*, and the various transgenic lines were grown under W or W + FR (Fig. S4). As expected, Col-0 hypocotyls strongly elongated in W + FR in contrast to the mild elongation of *pif7* (Fig. 3e–g). In W, hypocotyls of *pif7^PIF7wt^* seedlings were significantly longer than those of Col-0 and *pif7*, in agreement with published information (Li et al 2012; Chung et al 2020). In W + FR, *pif7^PIF7wt^* hypocotyls elongated, although less than Col-0 seedlings (Fig. 3e). In contrast, *pif7^PIF7Bm^* hypocotyls were similar to those of *pif7* seedlings in both W and W + FR (Fig. 3f), suggesting that the bHLH-defective *PIF7Bm* transgene had no biological activity. In W, hypocotyls of *pif7^PIF7APBm^* lines were longer than those of *pif7^PIF7WT^*, suggesting that *PIF7APBm* was more active than *PIF7wt* in promoting elongation in W. In W + FR, *pif7^PIF7APBm^* lines also exhibited longer hypocotyls than *pif7^PIF7WT^* lines (Fig. 3g). Transient expression of GFP-fused versions in *Nicotiana benthamiana* leaves confirmed that both PIF7wt and PIF7APBm localized to the nucleus (Fig. S5), suggesting that the mutated APB domain has no apparent impact on the subcellular location of the PIF7 variant.

To more clearly establish whether the activity of transgenic *PIF7* derivatives was enhanced in response to shade, the shade-induced hypocotyl elongation was calculated (ie difference in hypocotyl length of W + FR and W, HYP_W_  _+_  _FR_-HYP_W_) (Fig. 3h–j). As expected, this difference was maximum in Col-0 (ca. 6 mm) and minimum in *pif7* (1.5 to 2.0 mm). Transgenic *pif7^PIF7wt^* lines elongated more in response to simulated shade than *pif7*, suggesting that PIF7wt activity was enhanced by W + FR. By contrast, this difference in *pif7^PIF7Bm^* and *pif7^PIF7APBm^* lines was similar to that of *pif7* seedlings (Fig. 3i and j), indicating that the activity of both derivatives was not enhanced (ie did not change) in response to shade. As PIF7APBm was active in promoting growth, the interaction with active phyB appears to require an increase in PIF7 activity in a shade-dependent manner. By contrast, the observation that the length of *pif7^PIF7Bm^* seedlings was indistinguishable from the untransformed *pif7* parental line under W and W + FR strongly suggests that the loss of bHLH functionality in the PIF7Bm variant renders the protein inactive.

To confirm whether the lack of PIF7 activity in *pif7^PIF7Bm^* lines was not due to the absence of PIF7Bm protein, immunoblot analyses were carried out using an anti-HA antibody. These experiments detected bands of similar intensity corresponding to the PIF7 protein in both *pif7^PIF7wt^* and *pif7^PIF7Bm^* seedlings (Fig. S6), leading to the conclusion that PIF7Bm was indeed produced. We also noticed that PIF7APBm accumulated to much higher levels than PIF7wt protein (Fig. S6), which suggested that PIF7APBm is more stable than the other 2 PIF7 derivatives. One possible interpretation is that interaction with active phyB in the nucleus regulates PIF7 abundance.

### Abundance of the PIF7wt and PIF7Bm proteins is more sensitive to shade than PIF7APBm

**Figure 4:**
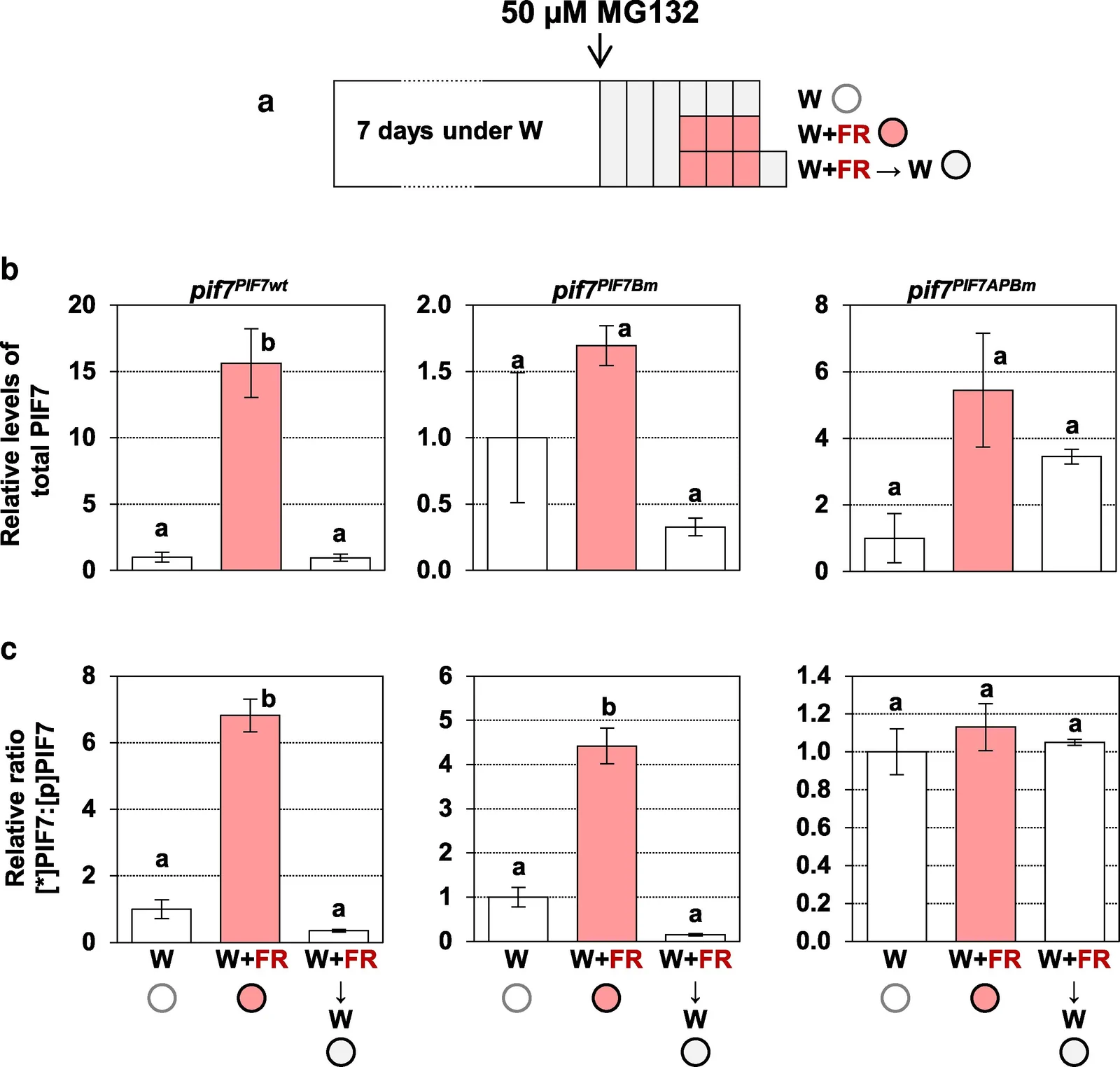
PIF7APBm shows a distinct phosphorylation pattern in response to low R:FR compared with the PIF7wt and PIF7Bm variants. a) Cartoon representing the experimental design. W-grown 7-d-old seedlings were transferred to a 50-µM MG132 solution for 3 h. Next, seedlings were kept under W for 3 h, transferred to W + FR for 3 h, or transferred to W + FR for 3 h and then moved back to W for 1 h. b) Immunoblot analyses were performed to quantify all PIF7-3xHA bands in seedlings of *pif7^PIF7wt^*, *pif7^PIF7Bm^*, and *pif7^PIF7APBm^* lines. Total PIF7-3xHA signal was normalized to ACTIN levels. c) Relative ratio of [*]PIF7:[p]PIF7 forms in the same extracts used in b. In b and c, data are the mean ± SE of 3 independent biological replicates and are expressed relative to the corresponding genotype in W. Different letters denote significant differences among means (1-way ANOVA with the Tukey test, *P* < 0.05).

The high accumulation of PIF7APBm in independent transgenic lines (Fig. S6) prompted an analysis of shade-dependent stability among the 3 variants. Because PIF7wt and PIF7Bm accumulate at very low levels (Fig. S6), 7-d-old seedlings were treated with MG132, a 26S proteasome inhibitor that blocks degradation of photolabile PIFs (Shen et al 2007) (Fig. 4a). After 3 h of MG132 treatment, seedlings were kept in W for 3 h, transferred to W + FR for 3 h, or transferred to W + FR for 3 h and then shifted back to W for 1 h (W + FR → W) (Fig. 4a). Although MG132 treatment only moderately increases PIF7 abundance (Chung et al 2020), it enabled clearer detection of PIF7wt and PIF7Bm in immunoblot assays (Fig. S7a–c), but PIF7APBm still accumulated to higher levels than PIF7wt and PIF7Bm under MG132 treatment (Fig. S7d). The abundance of PIF7wt, but not that of PIFBm, increased in response to W + FR relative to those in W or W + FR → W conditions (Fig. 4b, Fig. S7a–c), suggesting that PIF7 abundance shows some shade sensitivity (Li et al 2012). However, the large variability among independent biological replicates precluded reaching a clear conclusion in terms of the impact of shade on PIF7Bm and PIF7APBm protein levels (Fig. 4b, Fig. S7b and c).

The 2 bands detected with our commercial anti-HA antibody in *pif7^PIF7wt^*, *pif7^PIF7Bm^*, and *pif7^PIF7APBm^* extracts (Fig. S7a–c) likely correspond to the 2 forms described for wild-type PIF7: a slow-migrating phosphorylated form ([p]PIF7) and a fast-migrating dephosphorylated form ([*]PIF7), identified as the active form promoting shade-induced hypocotyl elongation (Li et al 2012; Huang et al 2018; Willige et al 2021; Martinez-Vasallo et al 2024). To estimate the proportion of these forms in our lines, we next calculated the relative ratio [*]PIF7:[p]PIF7 normalized to 1 (Fig. 4c). While the proportion of dephosphorylated PIF7 variants increased in *pif7^PIF7wt^* and *pif7^PIF7Bm^* seedlings when exposed to W + FR and decreased when illuminated with W, *pif7^PIF7APBm^* seedlings showed no change (Fig. 4c). These results indicate that light conditions (ie phyB activity) do not alter the proportion of dephosphorylated PIF7APBm protein, hence explaining why its activity is insensitive to shade. They also demonstrate that interaction with active phyB is crucial for regulating PIF7 activation via dephosphorylation.

### Both APB and bHLH domains are needed for the shade-induced regulation of *PIF7* target gene expression

**Figure 5:**
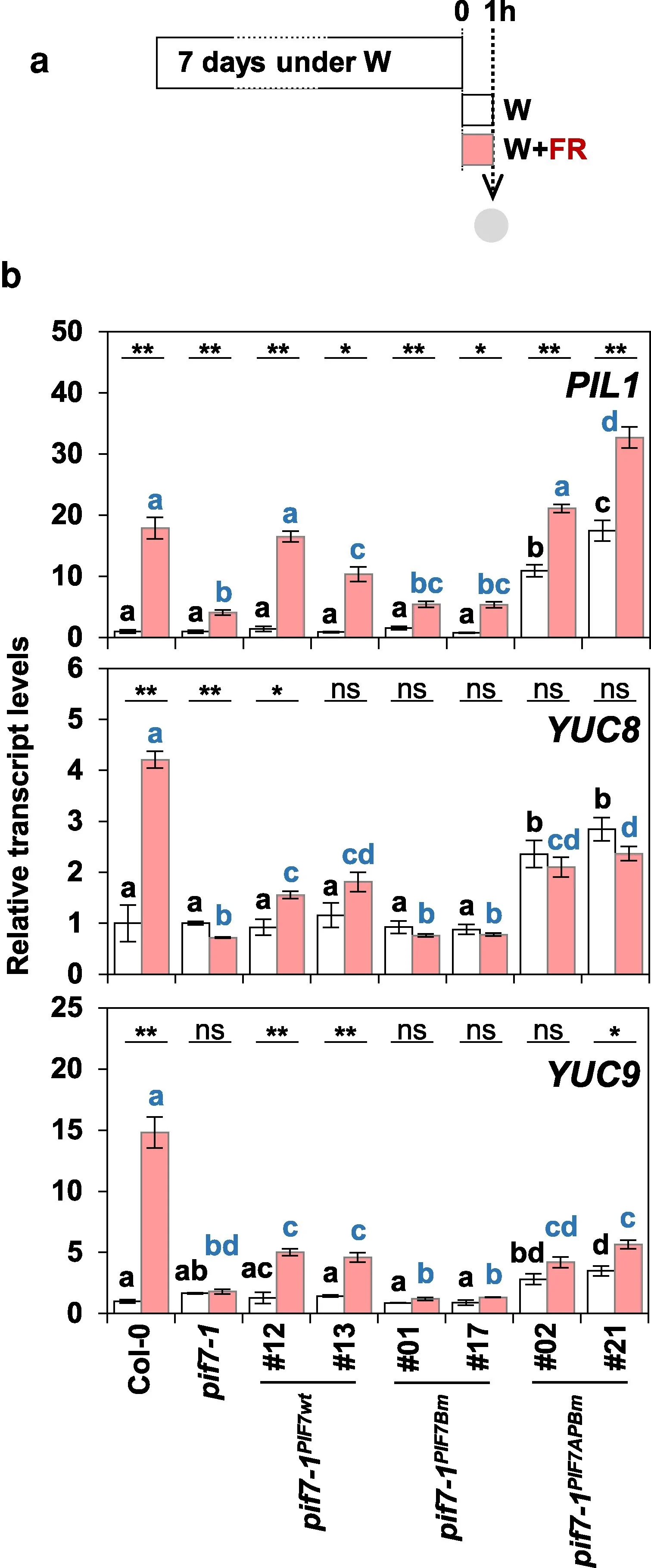
Mutant Arabidopsis PIF7 derivatives display different transcriptional activity in the early and rapid shade-regulated expression of PIF7 direct target genes in *A. thaliana* seedlings. a) Cartoon showing the experimental design. Seedlings were germinated and grown for 7 d in W and then maintained in W or transferred to simulated shade (W + FR) for 1 h before being harvested. b) Relative expression of *PIL1*, *YUC8*, and *YUC9* in seedlings of Col-0, *pif7-1*, *pif7^PIF7wt^*, *pif7^PIF7Bm^*, and *pif7^PIF7APBm^* lines in W and W + FR. Values are the mean ± SE of 3 independent biological replicates. Expression levels are shown relative to the Col-0 genotype in W. Black asterisks indicate significant differences between W and W + FR within each genotype (Student *t* test: **P < 0.01, *P < 0.05; ns, not significant). Different letters indicate significant differences among genotypes under W (black letters) or W + FR (blue letters) (1-way ANOVA with Tukey test, *P* < 0.05).

The primary molecular function of PIF7 is transcriptional regulation. Genome-wide analyses have identified PIF7 target genes in seedlings exposed to warm temperatures or shade (Chung et al 2020; Willige et al 2021). Exposure of *A. thaliana* wild-type seedlings to simulated shade triggers rapid induction of several direct target genes of PIF7, including *PIF3-LIKE 1* (*PIL1*), *YUCCA8* (*YUC8*), and *YUC9* (Hornitschek et al 2009; Li et al 2012). To assess the transcriptional activity of the PIF7 variants, 7-d-old W-grown seedlings were exposed for 1 h to W or W + FR before gene expression analyses (Fig. 5a). Expression of *PIL1*, *YUC8*, and *YUC9* was strongly induced by shade in Col-0 but not in *pif7* mutant seedlings (Fig. 5b), as reported (Li et al 2012; Jiang et al 2019).

The 3 PIF7 derivatives exhibited distinct transcriptional behavior. In *pif7^PIF7Bm^* seedlings, expression of the 3 genes was comparable to that in *pif7*, consistent with the loss of biological activity deduced from phenotypical and immunoblot analyses (Fig. 3, Fig. S6). These results are consistent with the E175D mutation abolishing the DNA-binding capacity of PIF7.

In W, transcript levels of *PIL1*, *YUC8*, and *YUC9* were similar in Col-0, *pif7*, and *pif7^PIF7wt^* seedlings but markedly elevated in *pif7^PIF7APBm^* seedlings (*PIL1*, 10- to 15-fold; *YUC8*, 2- to 3-fold; *YUC9*, 3- to 4-fold) (Fig. 5b). Following W + FR, *PIL1* expression was strongly induced in Col-0 (18-fold) and mildly in *pif7* (4-fold) seedlings. In *pif7^PIF7wt^* seedlings, *PIL1* expression reached near wild-type levels, and its expression was further induced in response to shade in *pif7^PIF7APBm^* seedlings (Fig. 5b). Shade treatment significantly increased *YUC8* and *YUC9* expression in Col-0 but not in *pif7*, in which *YUC8* was even repressed, or in *pif7^PIF7Bm^*. Both *YUC* genes were shade-induced in *pif7^PIF7wt^* seedlings, although to a lower extent than in Col-0, while their expression in *pif7^PIF7APBm^* remained high and unresponsive to shade (Fig. 5b). Together, these results indicated that (i) PIF7wt partially complemented the *pif7-1* mutant phenotype, (ii) PIF7Bm was inactive, and (iii) PIF7APBm displayed constitutive and even shade-independent transcriptional activity.

### PIF7APBm binds to DNA in both W and W + FR conditions

**Figure 6:**
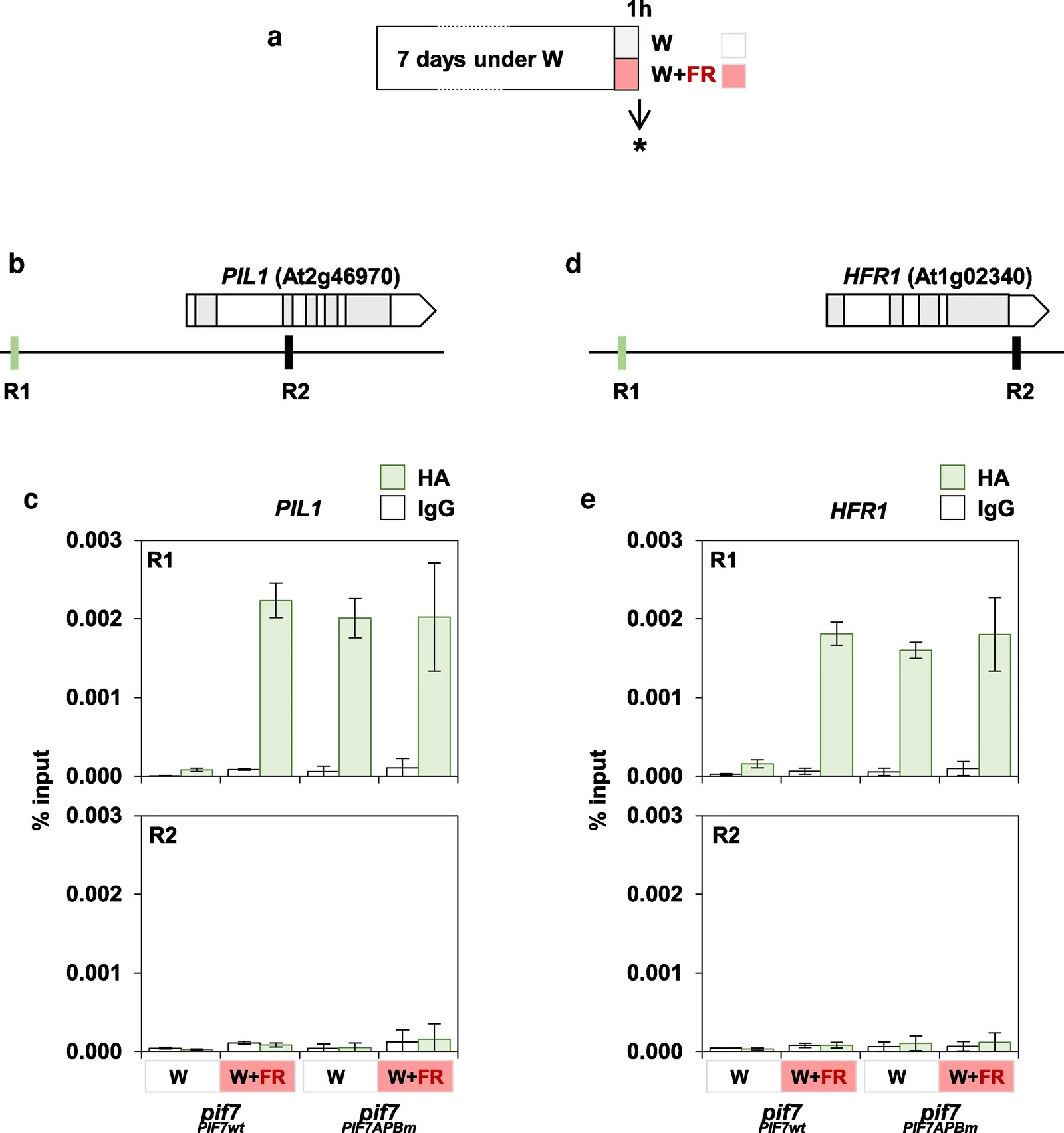
PIF7APBm binds to *PIL1* and *HFR1* gene promoters in both W and W + FR conditions. a) Cartoon representing the design of the experiment. W-grown 7-d-old seedlings were kept under W or W + FR for 1 h before harvesting. b and d) Schematic location of the 2 ChIP-qPCR amplified regions in b) *PIL1* and d) *HFR1* genes, R1 and R2. In both genes, only R1 (light green box) contains the G-boxes bound by PIF7. c and e) Percentage of input in R1 and R2 after ChIP of c) *PIL1* and e) *HFR1* genomic regions with anti-HA and anti-IgG antibodies is shown for each genotype and treatment. Values are means and bars are the SD of 3 biological replicates.

PIF7 binding to the promoters of its target genes occurs predominantly under shade, when phyB-dependent inhibition is relieved, leading to PIF7 dephosphorylation, whereas only weak or no DNA binding is detected under high R:FR (Li et al 2012; Willige et al 2021; Burko et al 2022). Given that light/shade conditions also affect chromatin structure (Martinez-Garcia and Moreno-Romero 2020), we aimed to explore whether the shade-independent transcriptional activity of PIF7APBm was due to constitutive binding to its target gene promoters and/or changes in chromatin environment that affected the accessibility of PIF7 to its binding sites. Chromatin immunoprecipitation (ChIP) assays were performed in *pif7^PIF7wt^* and *pif7^PIF7APBm^* seedlings grown under W or W + FR (Fig. 6a), focusing on *the* well-established PIF7 target genes *PIL1* and *HFR1*. Analysis of the *R1* regions of both promoters, which contain the G-box sequences bound by PIFs, revealed significant enrichment in *pif7^PIF7wt^* samples grown under W + FR but not under W, as reported. By contrast, the PIF7APBm variant retained robust DNA-binding activity under both W and W + FR treatments (Fig. 6). No enrichment was detected in the negative control *R2* regions within the *PIL1* and *HFR1* gene bodies, with signal levels comparable to those obtained with the IgG control (Fig. 6c and e). These results are consistent with the observed shade-dependent and shade-independent biological activities of PIF7wt and PIF7APBm, respectively (Fig. 3).

## Discussion

PIF transcription factors are central regulators of the SAS responses in *A. thaliana*. Among them, genetic analyses have established PIF7 as the primary regulator of shade-induced hypocotyl elongation, whereas the PIFQ members make comparatively minor contributions (Lorrain et al 2008; Li et al 2012). Understanding the specific molecular mechanisms underlying PIF7 function is therefore essential for elucidating how SAS is modulated at the molecular level.

Structure–function analyses have been commonly used for mechanistic studies of other transcriptional regulators, including PIFs (Al-Sady et al 2008; Galstyan et al 2011, 2012; Gallemi et al 2017). Like other PIFs, PIF7 contains an APB and a bHLH domain, suggesting that phyB interaction is functionally linked to its transcriptional activity. However, studies of the photolabile PIF3, the founder member of the PIF family (Ni et al 1998; Martinez-Garcia et al 2000), revealed that its phyA/phyB-binding and DNA-binding activities are uncoupled (Al-Sady et al 2008). Specifically, phyA/phyB interaction is dispensable for the rapid light-dependent upregulation of *ELIP2*, a direct PIF3 target (Pfeiffer et al 2014), whereas DNA binding is not required for the long-term PIF3-dependent promotion of hypocotyl length (Al-Sady et al 2008). For PIF1, only DNA binding was shown to be required for long-term light-mediated inhibition of hypocotyl length (phyA/phyB binding was not tested) (Shen et al 2008). Whether these functions are uncoupled remains unresolved due to the lack of parallel analyses on rapid changes in gene expression on PIF1 target genes.

A similar separation of molecular functions has been observed for other regulators of shade signaling, such as ATHB4, a homeodomain-leucine zipper transcription factor whose protein–protein interaction activity is required for regulating both leaf polarity and seedling responses to vegetation proximity, while its DNA-binding activity contributes exclusively to leaf polarity (Sorin et al 2009; Gallemi et al 2017).

**Figure 7:**
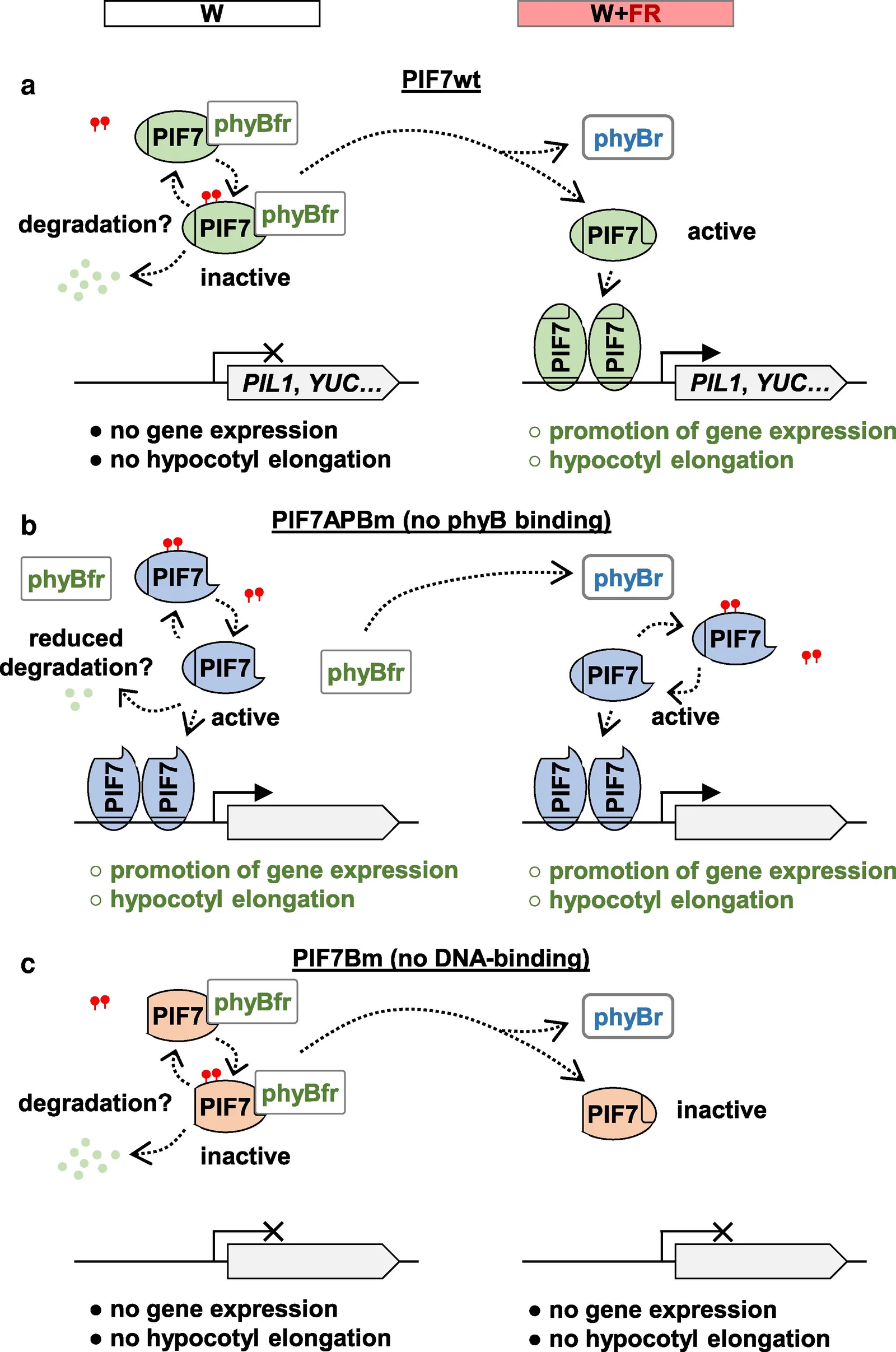
Model summarizing how phyB and DNA binding shape PIF7-mediated responses. a) Under W (high R:FR, left), phyB is predominantly active (phyBfr) and binds the wild-type form of PIF7, maintaining it in a phosphorylated (red tags), relatively unstable (?), and transcriptionally inactive state. Shade (low R:FR, right) inactivates phyB (phyBr), releasing PIF7, which becomes dephosphorylated, likely stabilized and transcriptionally active, enabling DNA binding, regulation of target gene expression, and promotion of hypocotyl elongation. b) The PIF7APBm variant cannot interact with phyB, so the balance between phosphorylated and dephosphorylated forms does not change; it is more stable and active under W, binds DNA, induces gene expression, and promotes hypocotyl elongation irrespective of the R:FR conditions. c) The PIF7Bm variant behaves like PIF7wt under W and W + FR but cannot bind DNA, preventing target gene regulation and hypocotyl elongation under shade. Therefore, it shows no biological activity in any R:FR condition.

For PIF7, it remained unknown whether such a functional separation is conserved (ie whether the APB domain can modulate hypocotyl growth independently of DNA binding). Our structure–function analyses demonstrate that DNA binding is essential not only for the rapid, shade-induced transcriptional upregulation of *PIL1*, *YUC8*, and *YUC9*, 3 well-characterized direct targets of PIF7, but also for the long-term promotion of hypocotyl length under shade (Figs. 3 and 5). The complete loss of all tested biological activities in the DNA-binding mutant PIF7Bm indicates that, unlike PIF3 (Al-Sady et al 2008), PIF7 activity fundamentally depends on DNA binding. Collectively, these results reveal that the phyB- and DNA-binding activities of PIF7 are mechanistically coupled, defining a distinct mode of action compared with PIF3. Indeed, the APB domain appears not to be required for either short-term transcriptional or long-term developmental outputs per se, but rather to confer shade-dependent regulation to both (Fig. 7).

While PIF7wt did not fully complement the *pif7* mutant, differences between the transgene and endogenous gene expression patterns (ie due to the use of the ∼2-kbp promoter fragment and/or the absence of introns in the transgene constructs, which might affect spatial and/or temporal expression and/or posttranscriptional regulation), rather than differences in expression levels or the protein themselves, are the most likely explanation for the incomplete complementation.

Although PIF7 is considered photostable compared with the highly photolabile PIFQ members, our analysis of the contrasting accumulation patterns of the PIF7wt and PIF7Bm proteins led us to conclude that its abundance exhibits a modest and somewhat variable light dependence (Fig. 4). Indeed, published studies have reported inconsistent findings regarding the effect of shade on PIF7 abundance (Li et al 2012; Willige et al 2021; Zhou et al 2021; Burko et al 2022). By contrast, the PIF7APBm variant accumulates at consistently high levels irrespective of light conditions (Fig. 4b, Fig. S7d), indicating that the APB domain confers a phyB-dependent regulation of protein abundance. Importantly, interaction with active phyB also governs PIF phosphorylation, increasing the proportion of the active dephosphorylated [*]PIF7 form (Fig. 4c). Consequently, loss of APB function results in a constant ratio of phosphorylated to dephosphorylated species (Fig. 4c) and likely stabilizes the protein over time under both W and W + FR (Fig. S6c). Conversely, the presence of a functional APB domain in PIF7Bm may prevent its accumulation (Fig. S6b) to levels sufficient for it to act as a dominant-negative regulator of PIFs. Together, these results indicate that interaction with active phyB exerts dual control over PIF7 molecular activity by modulating both its stability and its phosphorylation state (Fig. 7). Moreover, our data show that DNA binding is not required for the shade-induced shift in PIF7 phosphorylation (Fig. 4c).

Although both PIF7wt and PIF7APBm forms appear to have the same subcellular location (Fig. S5), PIF7wt transcriptional activity, but not PIF7APBm, is low under W, causing reduced DNA binding (Fig. 6) (Li et al 2012; Willige et al 2021). The constitutive binding of PIF7APBm to the *PIL1* and *HFR1* promoters under both W and W + FR (Fig. 6) highlights the crucial role of phyB interaction in modulating PIF7 function. It seems likely that when this interaction is lost, high nuclear accumulation of PIF7APBm maintains elevated levels of active [*]PIF7, leading to constitutive transcriptional activation of PIF7 direct target genes even under W conditions and, eventually, long hypocotyls (Figs. 3 and 5). The shade-independent activity of PIF7APBm in binding DNA is consistent with the finding that most PIF7 binding sites are located on permanently accessible chromatin (regardless of light condition) (Paulisic et al 2025). Therefore, chromatin accessibility does not appear to prevent PIF7 to bind its target DNA sequences under W.

Although *A. thaliana* PIF7 is the main regulator of shade-induced hypocotyl elongation, the extent to which this role is conserved in other plant species remains poorly studied. In tomato, the *SlPIF8a* gene was reported to play a major role in low R:FR responses, whereas *SlPIF4*, *SlPIF7a*, and *SlPIF7b* were found to have minor contributions, as *slpif4 slpif7a slpif7b* triple mutant plants elongate and respond similarly to the M82 (wild-type) seedlings (Rosado et al 2016; Kunta et al 2025). The weak expression of *SlPIF7a* and *SlPIF7b* may underlie their reduced involvement in the low R:FR responses (Kunta et al 2025). In contrast with these results, we observed attenuated elongation in *slpif7a slpif7b* double mutants compared to the MT (wild-type) parental line in both W and W + FR, although no role was found in the elongation response specifically induced by shade (Fig. 1, Fig. S2). Although variation in W + FR conditions might have an impact, the different genetic backgrounds between the *slpif4 slpif7a slpif7b* triple (M82) and *slpif7a slpif7b* double (MT) mutants might also strongly contribute to explaining the observed discrepancy. Indeed, in our simulated shade conditions, not only was Money Maker (MM) much more responsive than MT accession, but the shade response of most landraces was exacerbated compared to M82 (Burbano-Erazo et al 2025).

AtPIF7, together with the AtPIFQ members, also mediates hypocotyl elongation under short photoperiods (under high R:FR), conferring daylength sensitivity to optimize growth in the appropriate season (Leivar et al 2020). Our genetic analyses support that *SlPIF7a*, *SlPIF7b* (Fig. 1d, Fig. S2), and *ChPIF7* (Fig. 2, Fig. S3) also have a role in controlling hypocotyl length under high R:FR (no shade) (ie that these *PIF7* genes have retained this function). In addition, our analyses in *C. hirsuta*, a close relative of *A. thaliana* (Molina-Contreras et al 2019), also support a conserved role for *PIF7* in regulating plant responses to vegetation proximity across different plant species, at least when shade responses are not suppressed (Fig. 2). Phosphorylation of AtPIF7 is altered during the light hours in short-day photoperiods and at night (Leivar et al 2020). Together, these observations suggest that some of the mechanistic insights derived from our structure–function analyses of AtPIF7 can be extended to its orthologs in other species.

Because *C. hirsuta* exhibits a shade-tolerant growth habit, our findings support the notion that these plants have retained the core design of the SAS signaling pathway along evolution but modulated its strength (Martinez-Garcia and Rodriguez-Concepcion 2023). However, the activity of the additional mechanisms of control has been adjusted to optimize the response. Attenuation of the shade-induced elongation response results from enhanced activity of negative regulators, such as phyA and HFR1, or reduced activity of positive regulators such as PIF7 (Martinez-Garcia and Rodriguez-Concepcion 2023). Evolutionary tuning of the expression (and activity) of these components likely underpins the divergence between the shade-avoider *A. thaliana* and the shade-tolerant *C. hirsuta* and may mirror the human-driven selection of distinct *SlPIF7* functions observed in tomato cultivars M82 and MT.

## Experimental procedures

### Plant material and growth conditions

*A. thaliana* Col-0 and *pif7-1* (Leivar et al 2008; Li et al 2012) were used in this study to generate transgenic lines. *C. hirsuta* wild type (Oxford ecotype, Ox) and *sis1* (Molina-Contreras et al 2019) and tomato (*Solanum lycopersicum* L.) MT (Burbano-Erazo et al 2025) plants have been described before. To produce seeds, plants were grown in the greenhouse under LD photoperiod conditions (16 h W, 8 h darkness) at 22 °C, as described for *A. thaliana* (Martínez-Garcia et al 2014).

For *A. thaliana* and *C. hirsuta* hypocotyl assays, seeds were surface-sterilized and sown on solid growth medium without sucrose [0.5xGM–; 0.215% (w/v) MS salts plus vitamins, 0.025% (w/v) MES pH 5.8]. After 3 to 6 d of stratification, plates were incubated either in growth chambers at 22 °C under continuous white light (W) provided by either 4 cool-white vertical fluorescence tubes for 2 d (photosynthetic photon flux density in the PAR region, PPFD, of 20 to 25 µmol·m^−2^·s^−1^, R:FR > 3.3; light spectrum shown in Fig. S2 in Molina-Contreras et al 2019) or a dedicated growth chamber (model D1200PL; Aralab) at 22 °C and continuous W emitted from LED light tubes (PPFD of 55 µmol·m^−2^·s^−1^, R:FR = 3.55; light spectrum shown in Fig. S1 in Burbano-Erazo et al 2025). After that, plates were maintained in W or transferred to simulated shade conditions (W + FR). Simulated shade was generated by supplementing W with FR provided by 4 horizontal LED lamps (Philips GreenPower LED module HF far-red tubes). Growth chamber with W provided by fluorescent tubes (PPFD of 20 to 25 µmol·m^−2^·s^−1^) resulted in an R:FR of about 0.07.

For tomato seedling assays, seeds of tomato MT (wild type) and the 3 mutant lines were sown on solid 0.5xGM– and kept in the dark for 4 d at 22 °C. Those evenly grown seedlings were transferred to soil and incubated under white light (W) for 2 additional days in a walk-in growth chamber under LD photoperiod conditions (PPFD of 150 ± 20 µmol·m^−2^·s^−1^ at 25 ± 1 °C during the day and 22 ± 1 °C during the night/dark period) (Burbano-Erazo et al 2025). Then, half were kept under W and the other half transferred to W + FR for 11 d. W was obtained using a mix of 5 Philips MAS LEDtube 1,500 mm HO 23W840 and 4 MAS LEDtube 1,500 mm HO 23W830 LED tubes, arranged in an alternating pattern. To simulate proximity shade, FR provided by the Philips GreenPower LED module HF far-red tubes were added between the racks of LED tubes supplying W. As a result, in the same chamber, there was an area illuminated with W (R:FR of 3.14) and another area illuminated with W + FR (simulated shade, R:FR of 0.17) separated by an opaque panel, so W-grown plants were not exposed to residual FR. W and W + FR light spectra of this chamber are shown in Fig. S1 in Burbano-Erazo et al (2025).

For elongation assays, seedlings were laid down at the end of the experiment on a flat surface and pictures were taken. Hypocotyl measurements were carried out using the National Institutes of Health (NIH) ImageJ software. In *A. thaliana* and *C. hirsuta* experiments, 3 biological replicates (each biological replicate corresponded to ∼25 seedlings per genotype and treatment) were done. In tomato experiments, 16 to 21 seedlings per genotype and treatment were analyzed.

For gene expression analyses, immunoblot assays and chromatin immunoprecipitation, seeds were sown over a sterilized nylon membrane located on the top of the solid agar plates.

### Isolation of PIF7 mutants of tomato

Double mutants in *SlPIF7a* (Solyc03g115540) and *SlPIF7b* (Solyc06g069600), named *slpif7a slpif7b*, were obtained in tomato cv. MT using CRISPR-Cas9 gene editing. The guide RNAs (gRNAs) targeting both *SlPIF7* genes were designed with CRISPR-P 2.0 (Liu et al 2017) and selected to avoid mismatches with other genes. Sequences are shown in Table S1. Both gRNA sequences were placed under the control of the U6 promoter.

Cloning was performed following the CRISPR-Cas mutagenesis method (Schiml et al 2016) utilizing Cas9 nucleases and paired nickases. For single guide RNA cloning, the pEn-Chimera vector (Schiml et al 2016) was employed, while transfer into a binary vector was carried out using the pDE-Cas9 plasmid, which confers kanamycin resistance (Barja et al 2021). Inserted DNA fragments in all constructs were verified through restriction enzyme analysis and sequencing. The final binary vector was inserted into *Agrobacterium tumefaciens* strain GV3101 via electroporation, and the transformation was done using tomato cotyledon explants. The in vitro regeneration was conducted according to published regeneration protocols (Ellul et al 2003). Finally, explants exhibiting root formation and capable of growing on medium containing kanamycin were selected for transfer to the growth chamber under W (PPFD, 150 ± 20 μmol m^−2^ s^−1^), LD photoperiod (16 hours W, 8 h D), with daytime temperatures at 25 ± 1 °C and nighttime temperatures at 22 ± 1 °C. The T1 population was genotyped by PCR using specific primers to amplify the insert and confirm the successful transformation with *CAS9* (Table S1). In the T2 generation, homozygous plants obtained through natural self-fertilization of T1 were selected. PCR amplification with gene-specific primers was performed to confirm the presence of the insert (PIF7a: PIF7a_genoCRISPR_F and PIF7a_genoCRISPR_R; PIF7b: PIF7b_genoCRISPR_F and PIF7b_genoCRISPR_R), and the amplified product was further validated by sequencing with nested primers (PIF7a: PIF7a_genoCRISPR_R_SEQ; PIF7b: PIF7b_genoCRISPR_R_SEQ) detailed in Table S1. Finally, homozygous double mutant *slpif7a slpif7b* lines free of the *CAS9* transgene were selected in T3.

### Isolation of PIF7 mutants of *C. hirsuta*

To obtain loss-of-function mutants of *ChPIF7* in *C. hirsuta* (named *chpif7*), we synthesized 2 gRNAs targeting *ChPIF7*, gRNAChPIF7_1 (5′-ATG-GAG-TGG-AAG-AGC-TAA-CC-3′) and gRNAChPIF7_2 (5′-GGC-GTG-ATA-GGA-TAA-ACC-AG-3′). gRNAChPIF7_1 and gRNAChPIF7_2 sequences were placed under the control of the *A. thaliana U3* (pU3) *U6* (pU6) promoters, respectively. Both fragments were flanked by the Gateway attB1 and attB2 recombination sites (IDT, https://eu.idtdna.com/pages) (attB1 < pU3:gRNAChPIF7_1 < attB2, attB1 < pU6:gRNAChPIF7_2 < attB2). The sequences were recombined with the vector pDONR207 using Gateway BP Clonase II (Invitrogen) to generate the entry vector pSP103 (attL1 < pU3:gRNAChPIF7_1 < attL2) and pSP104 (attL1 < pU6:gRNAChPIF7_2 < attL2). Fragment produced by digestion of pSP104 with *Xho*I + *Pst*I was introduced by ligation into the pSP103 vector digested with *Sal*I + *Pst*I, resulting in a pSP105 vector that contained both gRNAs under the corresponding promoter flanked by the Gateway attB1 and attB2 recombination sites (attL1 < pU3:gRNAChPIF7_1 < pU6:gRNAChPIF7_2 < attL2). In a recombination reaction of pSP105 with pDE-Cas9 (Fauser et al 2014) using Gateway LR Clonase II, a binary vector pSP106 was created (attB1 < pU3:gRNAChPIF7_1 < pU6:gRNAChPIF7_2 < attB2, Cas9). This vector contained the information to target *ChPIF7* and conferred resistance to Phosphinothricin (PPT) in plants and spectinomycin in bacteria. *A. tumefaciens* strain C58C1 (pGV2260) was transformed with pSP106 by electroporation, and colonies were selected on solid YEB medium with antibiotics, as indicated before.

The *sis1* (phyA-deficient) mutant plants of *C. hirsuta* (Molina-Contreras et al 2019) were transformed by floral dipping with pSP106, and resistant transgenic seedlings were selected on 0.5xGM– medium containing PPT (30 μg/mL). The T1 seedlings were PCR genotyped using primers MJO27 and MJO28 (Table S2) to detect the presence of the transgene. In the following T2 generation, lines with 1 T-DNA insertion were grown to maturity, and its descendants with a suppression of the *sis1* phenotype were reselected in shade. *PIF7* fragments around the gRNA target sequences were amplified by PCR using primers SEO3 + SEO4, which include both gRNAChPIF7_1 and gRNAChPIF7_2. These fragments were sequenced using primers SEO3, SPO64, SEO5, and SEO4 (Table S2). Three lines with mutations in the *ChPIF7* gene were selected: *chpif7-1* (line #036; 7-nucleotide deletion at position 557 of the *ChPIF7* ORF), *chpif7-2* (line #038; 1-nucleotide deletion at position 558 of the *ChPIF7* ORF), and *chpif7-3* (line #034; insertion of 1 nucleotide at position 30 of the *ChPIF7* ORF). These lines were backcrossed with ChWT to get single mutants of *ChPIF7* in a wild-type *SIS1* background. The wild-type and mutant alleles were genotyped by PCR using primers WQO6 + ASO41 and WQO7 + ASO41 (to discriminate the wild-type *PIF7* and mutant *chpif7-1* alleles, respectively), ASO37 + ASO41 and ASO46 + ASO41 (to discriminate the wild-type *PIF7* and mutant *chpif7-2* alleles, respectively), and ASO39 + ASO41 and ASO40 + ASO41 (to discriminate the wild-type *PIF7* and mutant *chpif7-3* alleles, respectively) (Table S2).

### Generation of PIF7 variants for *pif7-1* complementation

Wild-type *PIF7* coding DNA sequence was amplified with the primers JO414 and JO415 using Col-0 cDNA as a template, which generated a DNA fragment that removed the stop codon and introduced a *XhoI* site (*PIF7^no_STOP^*). This PCR product was subcloned in a selected orientation (ATG close to the *Xho*I site from the multiple cloning site) into PCRII-TOPO (Invitrogen) to generate pRA1. Selected colonies were sequenced to confirm their identity. Plasmid pRA1 was digested with *Xho*I, and *PIF7_wt_* was inserted into pSP55 (containing a *3xHA* tag) (Paulisic et al 2021), which was previously digested with *Sal*I and dephosphorylated, to give pRA2. This plasmid contained the wild-type *PIF7* ORF fused in frame with *3xHA* (*PIF7_WT_-3xHA*). Finally, pRA2 was digested with EcoRI and *PIF7_WT_-3xHA* was directionally cloned into pENTR3C to obtain pRA3. This plasmid contained *PIF7_WT_-3xHA*, flanked with attL1 and attL2 sites (attL1 < *PIF7_WT_-3xHA* < attL2).

Derivatives of *A. thaliana PIF7* with the APB (*PIF7_APBm_*) and basic (*PIF7_Bm_*) domains mutated were obtained by PCR-based site-directed mutagenesis using pRA3 as a template. To obtain *PIF7_APBm_-*3xHA, 2 fragments were amplified using MJO25 plus JO417 and JO416 plus MJO25 primer combinations, respectively. Both PCR fragments were mixed and amplified using the nested JO414 and SPO32 primers. The resulting DNA fragment contained a full-length PIF7 with the mutated APB domain fused to the 3xHA. This PCR product was cloned into PCRII-TOPO, generating the plasmid pRA4 (*PIF7_APBm_-3xHA*). Selected colonies were sequenced to confirm the presence of the 2-point mutations that substituted Glu 8 with Ala (E8A) and Gly 14 with Ala (G14A) in the APB domain.

To obtain *PIF7_Bm_*, 2 fragments were amplified using MJO25 plus JO419 and JO418 plus MJO25 primer combinations, respectively. As before, both PCR fragments were mixed and amplified using the nested JO414 and SPO32 primers. The resulting DNA fragment contained a full-length PIF7 with the mutated basic domain fused to 3xHA. This PCR product was cloned into PCRII-TOPO, generating the plasmid pRA5 (*PIF7_Bm_-3xHA*). Selected colonies were sequenced to confirm the presence of a point mutation that substituted Glu 175 with Asp (E175D) in the basic domain.

pRA4 and pRA5 were digested with *Eco*RI, and the inserts containing the *PIF7* derivatives were directionally cloned into pENTR3C to obtain pRA7 (attL1 < *PIF7_APBm_-3xHA* < attL2) and pRA8 (attL1 < *PIF7_Bm_-3xHA* < attL2), respectively. These plasmids contained the *PIF7* derivatives flanked with attL1 and attL2 sites. To express these PIF7 derivatives in plants, pRA3, pRA7, and pRA8 were digested with *Bam*HI and *Xho*I. The resulting *Bam*HI-*Xho*I fragments were subcloned into the *Bam*HI-*Sal*I digested pCAMBIA1300-based pCS14 vector (containing the CaMV 35S promoter, *p35S*) (Sorin et al 2009) to generate pBA18 (*p35S:PIF7_WT_-3xHA*), pBA6 (*p35S:PIF7_APBm_-3xHA*), and pBA16 (*p35S:PIF7_Bm_-3xHA*).

We amplified a ca. 2-kb fragment of *PIF7* promoter (*pPIF7*) starting immediately before the ATG of the *PIF7* gene using gDNA of *A. thaliana* wild-type Col-0 as a template and primers JDO14 and SPO20. This fragment was subcloned into pCRII-TOPO to generate pSP94. Selected colonies were sequenced to confirm their identity. Finally, pBA18, pBA6, and pBA16 were digested with *Eco*RI (which removed the p35S) and dephosphorylated, and the *Eco*RI fragment from pSP94 containing the pPIF7 was subcloned into the same site. The resulting binary vectors were named pPP8 (*pPIF7:PIF7_WT-_3xHA*), pPP5 (*pPIF7:PIF7_APBm_-3xHA*), and pPP6 (*pPIF7:PIF7_APBm_-3xHA*). These binary vectors confer resistance to kanamycin in bacteria and hygromycin in plants.

### Generation of transgenic lines

*A. thaliana pif7-1* plants were transformed with pPP8, pPP5, and pPP6 to express the different At*PIF7* derivatives. Transgenic seedlings were selected on 0.5xGM– medium with hygromycin (30 µg·mL^−1^) and verified by PCR genotyping using specific primers. Homozygous transgenic plants with 1 T-DNA insertion were finally used for the experiments. The obtained lines were named *pif7^PIF7WT^, pif7^PIF7Bm^*, and *pif7^PIF7APBm^*.

### Subcellular localization analyses

Confocal microscopy was performed in agroinfiltrated *N. benthamiana* leaves using a Zeiss LSM-780 confocal microscope. Constructs to express the 2 *PIF7* variants were generated as follows. The plasmids pRA3 and pRA7 (see above) were amplified using the primers MDO3 and MDO4 in order to add attB1 and attB2 to the sequences of PIF7wt and PIF7_APBm_. The PCR products were cloned into a pDONR207 plasmid, resulting in pMD1 (attB1 < *PIF7wt-3xHA* < attB2) and pMD3 (attB1 *<*  *PIF7_APBm_-3xHA* < attB2). Finally, pMD1 and pMD3 were recombined using the Gateway system with the pMDC43 plasmid, which has a 35S promoter that controls the expression of the insert. The resulting binary vectors were named pMD2 (*p35S:GFP-PIF7wt-3xHA*) and pMD4 (*p35S:GFP-PIF7_APBm_-3xHA*). These binary vectors confer resistance to kanamycin in bacteria and hygromycin in plants.

### RNA extraction and gene expression analyses

Seven-day-old seedlings grown under the specific conditions for each experiment were used to extract total RNA (each biological replicate corresponded to about 30 to 50 seedlings). The seedlings were harvested and frozen in liquid nitrogen. The RNA was extracted using commercial kits (Maxwell RSC Plant RNA kits; Promega) following the manufacturer’s protocol and quantified using a NanoDrop 8000 spectrophotometer (Thermo Fisher Scientific). In total, 2 µg total RNA was retrotranscribed to cDNA in a final volume of 20 µL by using the Transcriptor First Strand cDNA Synthesis Kit (Roche). Subsequently cDNA was diluted 10-fold with water and stored at −20 °C for further analysis.

Relative messenger RNA abundance was determined via real-time quantitative PCR (RT-qPCR) in a final volume of 10 µL made up of 0.3 µM of both forward and reverse primers, 5 µL LightCycler 480 SYBR Green I Master Mix (Roche), and 2 µL 10-fold diluted cDNA (Molina-Contreras et al 2019). RT-qPCR was carried out in a LightCycler 480 Real-Time PCR system (Roche). This analysis was performed with 3 independent biological replicates for each condition and 3 technical replicates for each biological replicate. *UBIQUITIN 10* (*UBQ10*) and *ELONGATION FACTOR 1α* (*EF1α*) were used as endogenous reference genes to normalize the expression of the genes of interest. The primers used for the RT-qPCR analyses are provided as supplemental information (Table S3).

### Protein extraction and immunoblot analyses

About 50 mg of 7-d-old seedlings (about 40 seedlings), grown as indicated in the corresponding experiments, were used to extract proteins (Gallemi et al 2017). Plant material was harvested, weighed, and frozen in liquid nitrogen until use. Plant material was ground to a powder, and total proteins were extracted using 1.5 µL Extraction Buffer [EB, 40 mM Tris-HCl, pH 6.8, 4% (w/v) SDS, 5% glycerol, 1× cOmplete, Mini, EDTA-free Protease Inhibitor Cocktail (PI); Merck], per milligram of fresh weight. Protein concentration was determined using the Pierce BCA Protein Assay Kit (Thermo Scientific). Proteins (45 to 50 µg) were resolved in a 12% SDS-PAGE gel and transferred to a PVDF membrane (Bio-Rad WET transference system or Trans-Blot Turbo Transfer System), immunoblotted with rat monoclonal anti-HA (high affinity, clone 3F10, 1:2,000 dilution; Roche). The membrane was immunoblotted again with rabbit antiactin (polyclonal, 1:5,000 dilution; Agrisera). Secondary antibodies used were horseradish peroxidase (HRP)–conjugated goat anti-rat (polyclonal, A9037, 1:5,000 dilution; Sigma) or HRP-conjugated donkey anti-rabbit (1:10,000 dilution; Amersham). Blot pictures were obtained with the ChemiDocTm Touch Imaging system (Bio-Rad) or Amersham ImageQuant800 (Cytiva) using ECL Prime Western Blotting Detection Reagent (GE Healthcare, RPN2236).

### Statistical analyses

Statistical analyses were carried out using the Real Statistics Resource Pack, an Excel add-in that extends Excel's standard statistics capabilities. The type of analyses and compared measurements are indicated in the figure legends.

### Chromatin immunoprecipitation protocol

Seven-day-old seedlings grown in W were transferred to W + FR for 1 h or kept in W. After that, plant material (400 seedlings per biological replicate) was harvested and submerged in crosslinking solution [1× PBS, 0.01% (w/v) Triton X-100, 1% (w/v) formaldehyde]. They were vacuum infiltrated for 16 min, and the crosslinking was vacuum quenched for 8 min by adding 2 M glycine to a final concentration of 0.125 M. Then, the tissue was rinsed with water, dried on filter paper, and stored at −80 °C until processing.

For nuclei extraction, samples were ground to a powder and mixed with MEB buffer [1 M hexylene glycol, 20 mM PIPES-KOH pH 7.6, 10 mM MgCl_2_, 0.1 mM EGTA pH 8.0, 20 mM NaCl, 60 mM KCl, 0.5% (w/v) Triton X-100; 5 mM β-mercaptoethanol, 1× PI] 15 min under rotation at 4 °C. The homogenate was filtered twice through Miracloth and centrifuged (6 min at 4 °C at 1,500 × *g*) to pellet the nuclei. After removing the supernatant, nuclei were resuspended in Nuclei Lysis Buffer [50 mM Tris-HCl pH 8.0, 10 mM EDTA, 1% (w/v) SDS; 1× PI]. Then, chromatin was sonicated for 10 × 20 s “ON,” 45 s “OFF” (high power) in a Bioruptor (Diagenode). Samples were then diluted 10 times with chromatin immunoprecipitation (ChIP) dilution buffer [1.1% (w/v) Triton X-100, 1.2 mM EDTA, 16.7 mM Tris-HCl pH 8.0, 167 mM NaCl, 1× PI] and centrifuged (5 min at 8,000 × *g*) to collect the sonicated chromatin in the supernatant. Equal amounts of chromatin solution were incubated overnight at 4 °C with an α-HA antibody (Abcam rabbit polyclonal to HA tag—ChIP grade, ab9110) or IgG, and 10 µL was kept as input. Dynabeads Protein A for Immunoprecipitation (Thermo Fisher Scientific) were equilibrated with ChIP dilution buffer and added to the chromatin solution (1/10 of the chromatin volume). Samples were rotated gently at 4 °C for 90 min. Afterward, the beads were captured with a magnetic rack, and the supernatant was discarded. The beads were washed a total of 4 times: twice with Low Salt Buffer [150 mM NaCl, 0.1% (w/v) SDS, 1% (w/v) Triton X-100, 2 mM EDTA, 20 mM Tris-HCl pH 8.0] and twice with High Salt Buffer [500 mM NaCl, 0.1% (w/v) SDS, 1% (w/v) Triton X-100, 2 mM EDTA, 20 mM Tris-HCl pH 8.0]. Finally, the immunoprecipitated chromatin was washed once with Tris EDTA pH 8.0 (TE) buffer. The volume of the buffers utilized was the same volume of chromatin used for the ChIP. Input samples and the immunoprecipitated samples were de-crosslinked using the IPure kit (Diagenode), and DNA was purified following the instructions of the manufacturer. Samples were ready for qPCR quantification.

### Accession numbers

Sequence data from this article can be found in the Arabidopsis Information Resource (TAIR) (https://www.arabidopsis.org/), the *C. hirsuta* genetic and genomic resource (http://chi.mpipz.mpg.de/assembly) databases, and the tomato Sol Genomics Network database (https://solgenomics.net/) under the following accession numbers: *AtEF1a* (AT5G60390), *AtPIF7* (AT5G61270), *AtPIF8* (AT4G00050), *AtPIL1* (AT2G46970), *AtUBQ10* (AT4G05320), *AtYUC8* (AT4G28720), *AtYUC9* (AT1G04180), *ChPIF7* (CARHR275020), *ChPIF8* (CARHR248090 and CARHR248100), *SlPIF7a* (Solyc03g115540), *SlPIF7b* (Solyc06g069600), and *SlPIF8a* (Solyc01g090790).

## Supplementary Material

kiag602_Supplementary_Data

## Data Availability

All data are incorporated into the article and its online supplementary material.
