# Supplementary material for: DNA-binding activity of PIF7 links phytochrome B signaling to plant responses to vegetation proximity
Source: Plant Physiol. 2026 Aug 12;202(1):kiag602. doi: 10.1093/plphys/kiag602 (PMC13529829; doi:10.1093/plphys/kiag602)
Supplement: Supplementary file 1 [file kiag602_supplementary_data.zip › 260709 TableS1-S3_primers.pdf]

Supplementary Table S1. Primers to generate tomato CRISP-Cas9 mutant lines in *SlPIF7A* and *SlPIF7B* used in this work. Underlined sequences in primers refer to the target 20 nucleotide gRNA sequences in *SlPIF7* genes.

**Primer name**	**Gene**	**Sequence (5`→ 3`)**
sgSlPIF7a_F:	*SlPIF7A*	ATTGGGTTATGAACAGCTGCTGCT
sgSlPIF7a_R:	*SlPIF7A*	AAACAGCAGCAGCTGTTCATAACC
sgSlPIF7b_F:	*SlPIF7B*	ATTGCTGTCCACAACCAATCAGAG
sgSlPIF7b_R:	*SlPIF7B*	AAACCTCTGATTGGTTGTGGACAG
PIF7a_genoCRISPR_F	*SlPIF7A*	CGAAAGAACACATGGTACATC
PIF7a_genoCRISPR_R_SEQ	*SlPIF7A*	GTAATGTCAGAGCCAGGAC
PIF7a_genoCRISPR_R	*SlPIF7A*	GTAACTACTCCACTAGCTAGC
PIF7b_genoCRISPR_F	*SlPIF7B*	CATAGCTAATGTGGCAGTC
PIF7b_genoCRISPR_R_SEQ	*SlPIF7B*	GCGTAACTAGTGAGTTCATG
PIF7b_genoCRISPR_R	*SlPIF7B*	GCACCAAAACCAAACAAAAC
CAS9_F	*CAS9*	TCCCTCATCAGATCCACCTC
CAS9_R	*CAS9*	CTGAAACCTGAGCCTTCTGG

Supplementary Table S2. Primers used to subclone *AtPIF7* and *ChPIF7* in the various vectors used in this work.

**Gene or sequence**	**Primer name**	**Sequence (5`→ 3`)**
AtPIF7	JO414	TAACACATGTCGAATTATGGAG
AtPIF7	JO416	AGCGCTCACATGGGAAAATGCGCAACTAACCGTTCATGGTC
AtPIF7	JO417	GCCCATTTTCCCATGTGAGCGCTTTAACTCCATAATTCGAC
AtPIF7	JO418	CAACGAGTCCGATAGGAGACGGCGTGATAGGAT

AtPIF7	JO419	GCCGTCTCCTATCGGACTCGTTGTGAATCGC
AtPIF7	MDO3	GGGGACAAGTTTGTACAAAAAAGCAGGCTACATGTCGAA TTATGGAGTT
AtPIF7	MDO4	GGGGACCACTTTGTACAAGAAAGCTGGGTGTCAAGCGTA ATCTGGAACG
attL1/2	MJO25	GACTGATAGTGACCTGTTCG
attB1	MJO27	GGGGACAAGTTTGTACAAAAAAGCAGGCT
attB2	MJO28	GGGGACCACTTTGTACAAGAAAGCTGGGT
ChPIF7	SEO3	TCTCACGCTTCTGTCTTTGTC
ChPIF7	SEO4	ATCCGCCTGTAGCAATATCCG
ChPIF7	SEO5	GAAAAACGATTGTCTAAAATG
pAtPIF7	SPO20	CCTCTAGAGTGTTACTTAGGCCGC
pAtPIF7	JDO14	GGTCTAGATAATTGAAAGAGTACATTCAAATATC
3xHA tag	SPO32	GGCTCGAGTCAAGCGTAATCTGGA
ChPIF7	SPO64	TGCTCGTCCCCGTCGTCCAT
ChPIF7	WQO6	GTGATAGGATAAACCAGAG
ChPIF7	WQO7	GTGATAGGATAAAATGAGA
ChPIF7	ASO37	GGCGTGATAGGATAACCC
ChPIF7	ASO46	GGCGTGATAGGATAAGCA
ChPIF7	ASO39	TGGAGTGGAAGAGCTCAC
ChPIF7	ASO40	TGGAGTGGAAGAGCTCAA
ChPIF7	ASO41	TAGATCCATGTTCATTGTCTG

Supplementary Table S3. Primers used for qPCR analyses. Several of the following primers have been described elsewhere (Sorin et al. 2009; Cifuentes-Esquivel et al. 2013; Molina-Contreras et al. 2019).

Gene	Primer name	Sequence (5`→ 3`)
AtPIF7	BAO06	TGGCCACAGCGTCACTGCAA
	BAO07	TGCTCGTCCCCGTCGTCCAT
AtPIL1	BO87	GGAAGCAAAACCCTTAGCATCAT
	BO88	TCCATATAATCTTCATCTTTTAATTTTGGTTTA
AtYUC8	BO134	AATGGACGCGGTTAAGATCG
	BO135	CCCCTTGAGCGTTTCGTG
AtYUC9	BO136	GGCATGGAAGTCTCTCTTGATCTT
	BO137	CGGTAAAACATGAACCGAGCTT
AtUBQ10	BO40	AAATCTCGTCTCTGTTATGCTTAAGAAG
	BO41	TTTTACATGAAACGAAACATTGAACTT
AtEF1α	BO95	TGGTGTCAAGCAGATGATTTGC
	BO96	ATGAAGACACCTCCTTGATGATTTC
PIL1 R1	JRO62	AATGGGACCCACAATTAGGC
	JRO63	AACACAAAGGGGTGGATGAA
PIL1 R2	JRO64	CGCATGAACTTGTGTCTTCG
	JRO65	GCTGCTTGTCTTAGTTCTTTCACA
HFR1 R1	JRO66	TGAATCAAATGGGCCTGAA
	JRO67	ATAGCACCATGTCCATCACG
HFR1 R2	JRO68	ATGTGATTCGCCGGATTTAG
	JRO69	GGCGACATTAGGATTTGGAA

Cifuentes-Esquivel N, Bou-Torrent J, Galstyan A, Gallemí M, Sessa G, Salla Martret M, Roig-Villanova I, Ruberti I, Martínez-García JF (2013). The bHLH proteins BEE and BIM positively modulate the shade avoidance syndrome in Arabidopsis seedlings. *Plant J* 75: 989-1002.

Molina-Contreras MJ, Paulisic S, Then C, et al. (2019) Photoreceptor activity underlies contrasting shade responses in Cardamine and Arabidopsis. *Plant Cell* 31: 2649–2663

Sorin C, Salla-Martret M, Bou-Torrent J, Roig-Villanova I, Martinez-Garcia JF (2009) ATHB4 modulates hormone responses during shade avoidance. *Plant J* 59: 266–277
