# Supplementary material for: DNA-binding activity of PIF7 links phytochrome B signaling to plant responses to vegetation proximity
Source: Plant Physiol. 2026 Aug 12;202(1):kiag602. doi: 10.1093/plphys/kiag602 (PMC13529829; doi:10.1093/plphys/kiag602)
Supplement: Supplementary file 1 [file kiag602_supplementary_data.zip › Figures S1-S7_corrected.pdf]

Figure S1

A

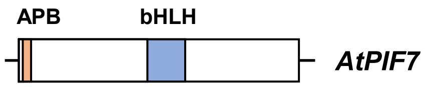

B **APB domain**

```
SlPIF7a  SKCEEVAELTWEKGQLGMHGLGG  68
SlPIF7b  NKC-EVAELTWENGQVAMHRLGS  24
AtPIF7   MSNYGVKELTWENGQLTVHGLGD  23
ChPIF7   MSNYGVEELTWENGQLTVHGLGD  23
SlPIF8a  SLDYEVAELTWENGQLAMHGLGP  56
AtPIF8   ILDYEVAELTWENGQLGLHGLGP  53
ChPIF8   MLDYEVAELTWENGQLGLHGLGP  57
              * *****:**: :* **
```

C **basic domain** **Helix-loop-helix**

```
                    5   9   13
                    •   •   •
SlPIF7a SSRRSRAAAVHNQSERRRRDRINQRMKALQRLVPNASK------TDKASMLDEVIDYLKQLQAQVQLMSS  291
SlPIF7b PSKRSRRAAVHNQSERRRRDRINEKMKALQKLVPNASKVLHILQTNKASMLEEVIKYLKQLQAQIQLISY  220
AtPIF7  NGRRGRAAAIHNESERRRRDRINQRMRTLQKLLPTASK------ADKVSILDDVIEHLKQLQAQVQFMSL  224
ChPIF7  NGRRGRAAAIHNESERKRRDRINQRMRTLQKLLPTATK------ADKVSILDDVIEHLKQLQAQVQFMSL  341
SlPIF8a STKRKRAAAIHNQSERKRRDKINQRMKTLQKLVPNSSK------TDKASMLDEVIEYLKQLQAQVHMMSR  271
AtPIF8  STKRSRAAAIHNQSERKRRDKINQRMKTLQKLVPNSSK------TDKASMLDEVIEYLKQLQAQVSMMSR  266
ChPIF8  STKRSRAAAIHNQSERKRRDKINQRMKTLQKLVPNSSK------TDKASMLDEVIEYLKQLQAQVSMMSR  272
          :* * **:**:**:***:**::*::**:*:*.::*      . .  ::.:**.:*******: ::*
```

Figure S1. PIF7 and PIF8 from *A. thaliana*, *C. hirsuta* and *Solanum lycopersicum* share conserved residues in the APB and bHLH domain. (A) Cartoon representing the location of the APB and bHLH domains in AtPIF7. Alignment of the region containing the (**B**) APB and **(C)** bHLH domains in SlPIF7a, SlPIF7b, AtPIF7, ChPIF7, SlPIF8a, AtPIF8 and ChPIF8. In **B**, light brown box indicates the location of the APB domain; residues in bold refer to those invariants in the various APB core sequences. In **C**, the light blue box indicates the limits of the bHLH domain; residues in bold refer to positions 5, 9, and 13 in the basic region, that correspond to the H-E-R conserved residues in the DNA-binding bHLHs.

Figure S2

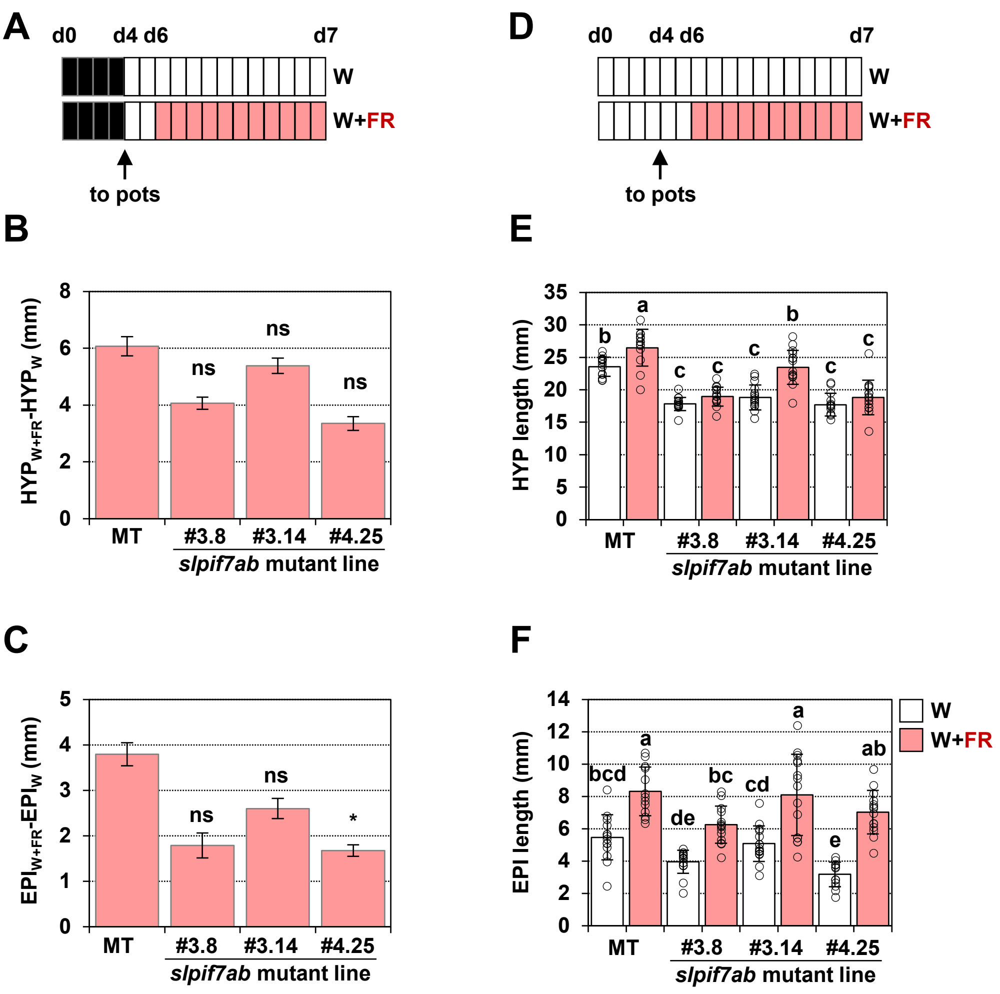

Figure S2. Seedlings of tomato mutant lines deficient in *PIF7* are shorter under both W and W+FR. (A) Cartoon showing the design of the experiment. Seedlings were germinated for 4 days in darkness, then seedlings of similar size were selected, transferred to individual pots. After keeping them for 2 days under long day photoperiodic conditions for acclimation, seedlings were either maintained in W or transferred to simulated shade (W+FR) for 11 more days. Elongation response of the **(B)** hypocotyl (HYP) and **(C)** epicotyl (EPI) (difference in HYP and EPI length in W+FR and W) of tomato lines shown in Figure 1D; error bars refer to propagated SE. Asterisk indicate significant differences (based on the two-way ANOVA shown in Figure 1D, ns, not significant) between the mutant and wild-type genotypes in response to simulated shade. **(D)** Cartoon showing the design of the experiment, which is similar to that shown in **A** except that seedlings were germinated for 4 days in the light. **(E)** HYP and **(F)** EPI length of MT and *slpif7ab* mutant lines grown under W (white bars) and W+FR (pink bars) conditions, as indicated in panel **D**. Values are the means ± SD of 16-21 individual seedlings per genotype and treatment (shown as empty black dots). Different letters above bars indicate statistically significant differences (one-way ANOVA with Tukey's test, $p<0.05$) among means.

Figure S3

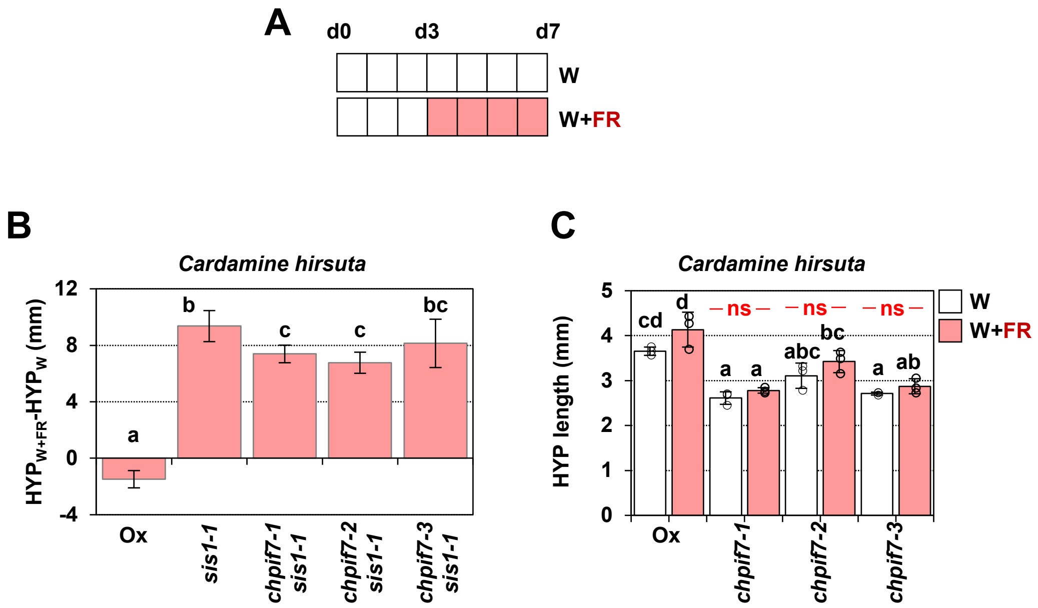

Figure S3. Seedlings of *C. hirsuta* mutant lines deficient in *PIF7* are shorter under both W and simulated shade. (A) Cartoon showing the experimental design. Seedlings were germinated for 3 days in W and then either maintained in W or transferred to simulated shade (W+FR) for 4 more days. **(B)** Elongation response (difference in hypocotyl (HYP) length in W+FR and W) of *C. hirsuta* lines shown in Figure 2B. Different letter denote significant differences (two-way ANOVA with Tukey test, P-value < 0.05) among means. **(C)** HYP length of Ox and *chpif7-1*, *chpif7-2* and *chpif7-3* single mutants. Values are the means ± SD of three independent biological replicates (shown as empty dots). Each biological replicate is the mean of ca. 25 seedlings. Different letters denote significant differences (one-way ANOVA with Tukey test, p<0.05) among means. Red asterisks/letters indicate significant differences (two way-ANOVA, p<0.05; ns, not significant) of the response to simulated shade of each single mutant *chpif7* line compared to Ox.

Figure S4

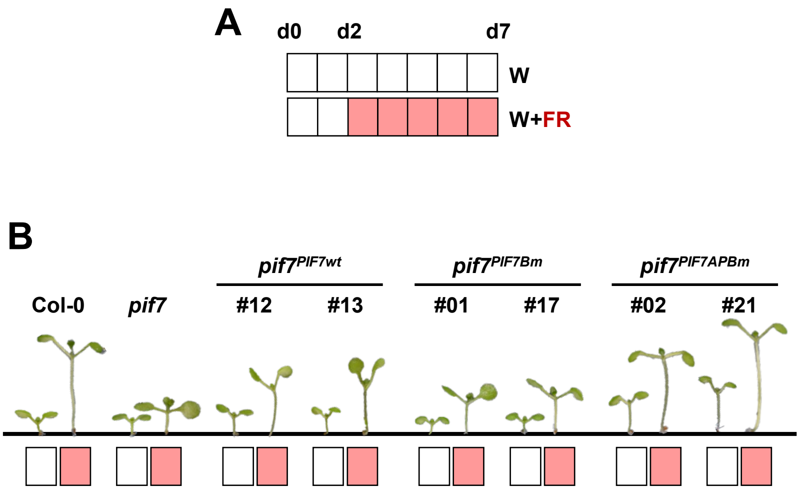

Figure S4. Aspect of representative *A. thaliana* seedlings of Col-0, *pif7-1*, $pif7^{PIF7wt}$, $pif7^{PIF7Bm}$ and $pif7^{PIF7APBm}$ grown in W and in W+FR. (A) Cartoon showing the design of the experiment. Seedlings were germinated and grown for 2 days in W and then either maintained in W or transferred to simulated shade (W+FR) for 5 more days. **(B)** Aspect of representative seedlings of the indicated lines shown in Figure 3 E-G.

Figure S5

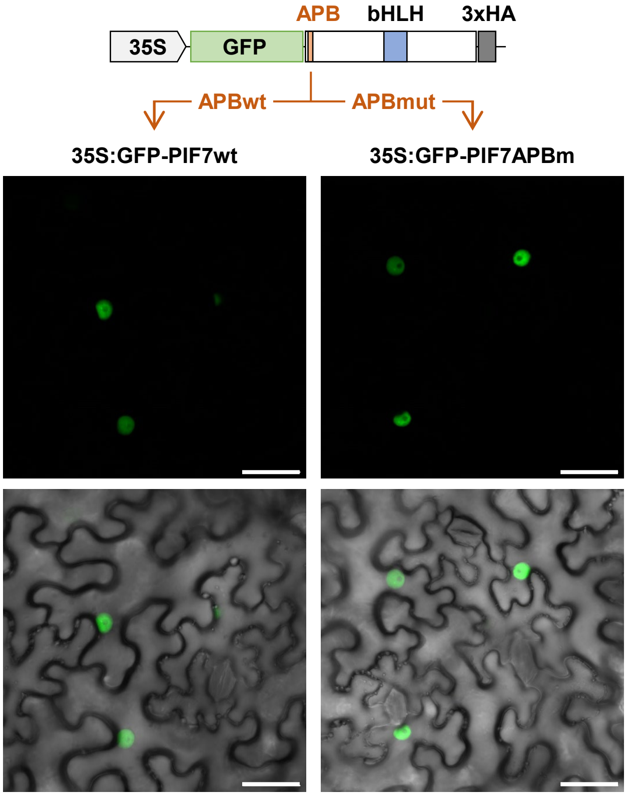

Figure S5. Both PIF7wt and PIF7APBm are nuclear proteins. Confocal images of cells of *Nicotinana benthamiana* leaves agroinfiltrated with GFP-PIF7wt (left) and GFP-PIF7APBm (right). The used constructs are illustrated above. Green fluorescence (top) and overlay of green fluorescence and bright-field (bottom) are shown. Images are a z-stack of 8 optical sections. Sale bars, 50 µm.

Figure S6

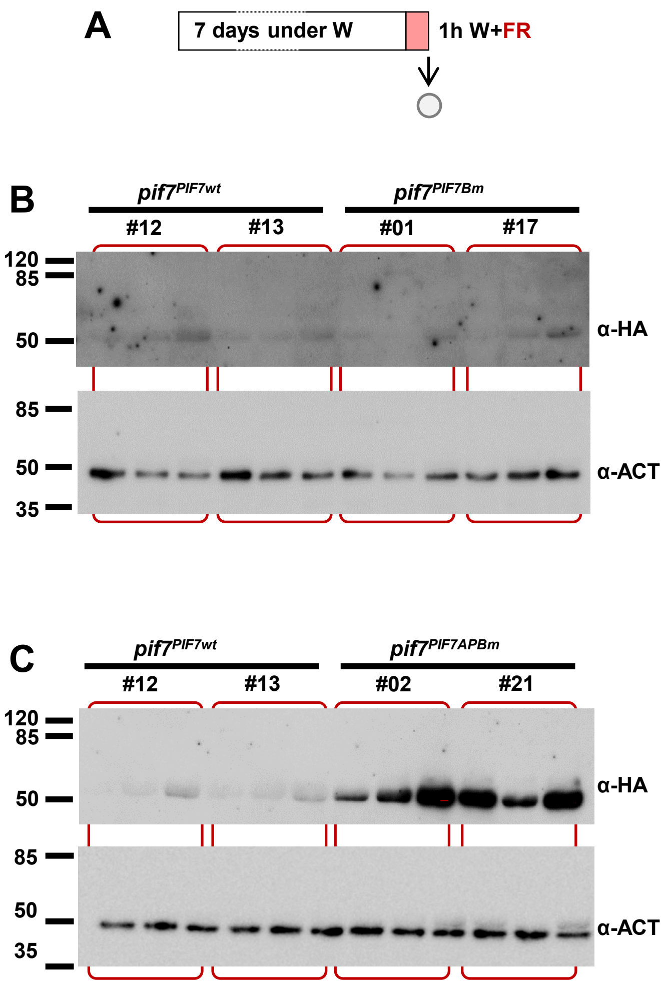

Figure S6. PIF7APBm accumulates to higher levels than PIF7wt and PIF7Bm proteins in transgenic *A. thaliana* seedling. (A) Cartoon illustrating the experimental design. Seedlings of *pif7^{PIF7wt}*, *pif7^{PIF7Bm}* and *pif7^{PIF7APBm}* lines were germinated and grown for 7 days in W and then transferred to W+FR for 1 h. Immunoblot analyses of PIF7 derivatives in *pif7^{PIF7wt}* and **(B)** *pif7^{PIF7Bm}* or **(C)** *pif7^{PIF7APBm}* lines. Three independent biological replicates are shown for each line. PIF7 derivatives were detected using an α-HA antibody (upper panels) and ACTIN was detected using an α-actin antibody (lower panels).

Figure S7

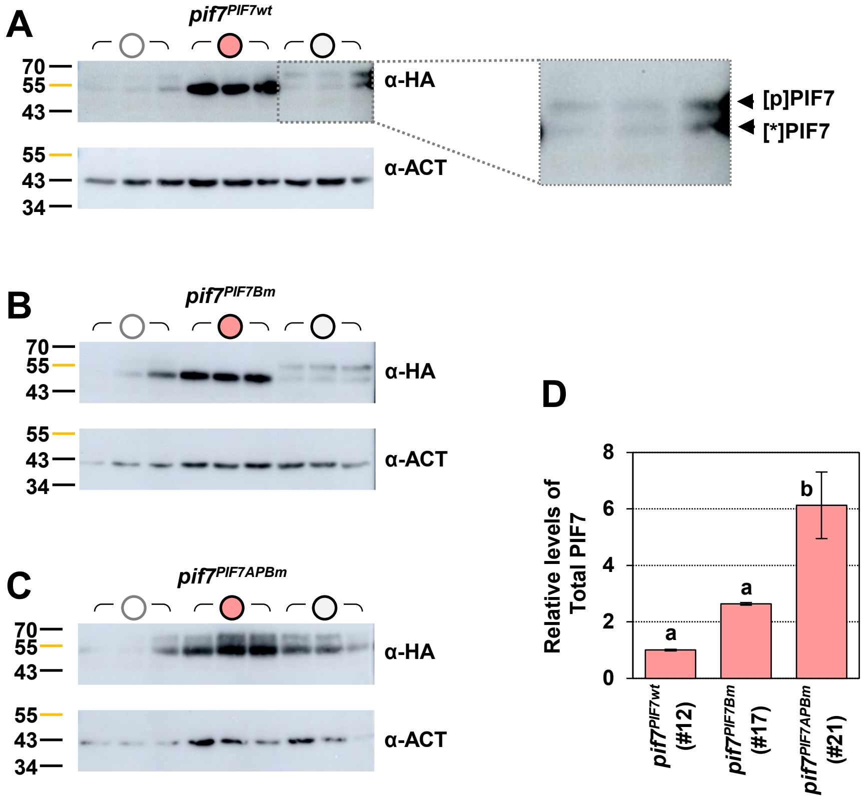

Figure S7. PIF7APBm accumulates to higher levels than PIF7wt and PIF7Bm derivatives. Immunoblot showing PIF7 derivatives in **(A)** *pif7^{PIF7wt}* (line 12) **(B)** *pif7^{PIF7Bm}* (line#17) and **(C)** *pif7^{PIF7APBm}* (line #21) seedlings. In each section, bands were detected in groups of consecutive 3 biological replicates with an anti-HA antibody (α-HA, upper panels) and an α-actin antibody (α-ACT, lower panels). Seedlings were grown as indicated in Figure 4A. Briefly, W-grown 7-day-old seedlings were transferred to a 50 µM MG132 solution for 3 h. Next, seedlings were either kept under W for 3 h, transferred to W+FR for 3 h, or transferred to W+FR for 3 and then moved back to W for 1 h. Inset in **A** shows the phosphorylated (upper, [p]PIF7) and non-phosphorylated (lower, [*]PIF7) forms of PIF7. **(D)** Relative PIF7-3xHA levels normalized to actin levels were estimated in seedlings of *pif7^{PIF7wt}*, *pif7^{PIF7Bm}* and *pif7^{PIF7APBm}* lines grown under W+FR. Data are means and error bars SE of three independent biological replicas. Values are relative to those of *pif7^{PIF7wt}*. Different letters denote significant differences (one-way ANOVA with the Tukey test, $p<0.05$) among means.
